# Rare missense mutations in *ABCA7* might increase Alzheimer’s disease risk by plasma membrane exclusion

**DOI:** 10.1186/s40478-022-01346-3

**Published:** 2022-03-31

**Authors:** Liene Bossaerts, Elisabeth Hendrickx Van de Craen, Rita Cacace, Bob Asselbergh, Christine Van Broeckhoven

**Affiliations:** 1 Neurodegenerative Brain Diseases Group, VIB Center for Molecular Neurology, Antwerp, Belgium; 2 Department of Biomedical Sciences, University of Antwerp, Antwerp, Belgium; 3 Department of Neurology, University Hospital Antwerp, Edegem, Belgium; 4 Neuromics Support Facility, VIB Center for Molecular Neurology, Antwerp, Belgium; 5 Department of Biomedical Sciences, VIB Center for Molecular Neurology, University of Antwerp - CDE, Universiteitsplein 1, 2610 Antwerp, Belgium

**Keywords:** Alzheimer’s disease, ABCA7, Missense mutations, Mislocalization

## Abstract

The adenosine triphosphate–binding cassette subfamily A member 7 gene (*ABCA7)* is associated with Alzheimer’s disease (AD) in large genome-wide association studies. Targeted sequencing of *ABCA7* suggests a role for rare premature termination codon (PTC) mutations in AD, with haploinsufficiency through nonsense-mediated mRNA decay as a plausible pathogenic mechanism. Since other classes of rare variants in *ABCA7* are poorly understood, we investigated the contribution and pathogenicity of rare missense, indel and splice variants in *ABCA7* in Belgian AD patient and control cohorts. We identified 8.36% rare variants in the patient cohort versus 6.05% in the control cohort. For 10 missense mutations identified in the Belgian cohort we analyzed the pathogenetic effect on protein localization in vitro using immunocytochemistry. Our results demonstrate that rare *ABCA7* missense mutations can contribute to AD by inducing protein mislocalization, resulting in a lack of functional protein at the plasma membrane. In one pedigree, a mislocalization-inducing missense mutation in *ABCA7* (p.G1820S) co-segregated with AD in an autosomal dominant inheritance pattern. Brain autopsy of six patient missense mutation carriers showed typical AD neuropathological characteristics including cerebral amyloid angiopathy type 1. Also, among the rare *ABCA7* missense mutations, we observed mutations that affect amino acid residues that are conserved in ABCA1 and ABCA4, of which some correspond to established *ABCA1* or *ABCA4* disease-causing mutations involved in Tangier or Stargardt disease.

**Supplementary Information:**

The online version contains supplementary material available at 10.1186/s40478-022-01346-3.

## Introduction

*ABCA7* was identified as a risk gene for late onset (≥ 65 years) AD through genome-wide association studies [1–6]. Since then, genetic studies have demonstrated that rare heterozygous premature termination codon (PTC) mutations in *ABCA7* are associated with AD risk in both early (< 65 years) and late onset AD patients [7–15]. Rare PTC mutations include nonsense, frameshift and canonical splice site mutations as well as the noncanonical c.5570 + 5G > C mutation, which leads to a stop codon due to aberrant splicing of exon 41 [11, 16]. Haploinsufficiency through nonsense-mediated mRNA decay (NMD) is suggested as a plausible downstream mechanism, although incomplete NMD and alternative splicing events, removing the PTC from the transcript, have been observed [11]. In a Belgian cohort, we previously identified PTC mutations in 4.87% of the AD patients compared to 1.84% of the control individuals [9]. In addition, a variable number of tandem repeats (VNTR) is present in intron 18 [17]. Expanded *ABCA7* VNTR alleles (> 5720 bp) are highly enriched in AD patients and correlate with reduced *ABCA7* expression and with increased exon 19 skipping [17]. However, the contribution to AD of other rare variants in *ABCA7,* e.g., missense, indels and noncanonical splice mutations, and their potential downstream pathogenic mechanism is less well investigated.

ABCA7 is involved in lipid metabolism and phagocytosis [18]. In vitro and in vivo studies suggest that ABCA7 deficiency leads to a decreased microglial Aβ clearance and elevated amyloid precursor protein (APP) processing, thereby exacerbating Aβ accumulation in the brain, a key pathological feature of AD [19–23]. ABCA7 belongs to the ATP-binding cassette (ABC) transporter family, a superfamily of highly conserved proteins responsible for the active transport of various substrates across cellular membranes [24]. ABC transporters share a characteristic architecture consisting of four core domains: two nucleotide binding domains (NBD) providing energy for substrate transport by ATP binding and hydrolysis, and two transmembrane domains (TMD) providing a pathway across the membrane for the transport of a substrate [25]. The A-subclass of ABC-transporters (ABCA) gained special interest since several members are causatively linked to monogenic diseases.

ABCA1 and ABCA4 show the most extensive sequence identity with ABCA7 (54% and 49%) [24]. Monoallelic mutations in *ABCA1* are the cause of familial high-density lipoprotein (HDL) deficiency, whereas homozygous or compound heterozygous mutations represent a more severe phenotype known as Tangier disease [26]. Bi-allelic mutations in *ABCA4* are responsible for various forms of recessive retinal dystrophies, including Stargardt disease (STGD1), while heterozygous mutations can increase disease risk or lead to later disease onset compared to bi-allelic mutation carriers [27–29]. The genetic spectrum of pathogenic *ABCA1* and *ABCA4* mutations is extensive, and missense mutations represent the largest group in both genes (http://www.hgmd.cf.ac.uk) [30]. Several studies demonstrated that disease-linked missense mutations in *ABCA1* and *ABCA4* affect subcellular localization and function, likely by inducing protein misfolding [31–37]. Also, missense mutations in regions of high sequence similarity between ABCA1 and ABCA4 show a similar impact on the protein in vitro*,* implying a similar structure–function relationship [35]. How mutations in *ABCA7* influence the subcellular protein localization or secretion is not known.

Studies analyzing the genetic contribution of *ABCA7* missense mutations to AD are limited, and conflicting results have been reported [11, 14, 16]. Additional studies reported missense mutation frequencies in AD patients and controls [8, 10, 38]. A recent meta-analysis showed a significant enrichment of *ABCA7* missense mutations in AD patients [39]. One study reported co-segregation of the ABCA7 missense mutation p.R880Q with autosomal dominant AD [40]. Earlier, we described the frequency and characteristics of *ABCA7* PTC mutation carriers in the Belgian AD patient and control cohort [9]. In this study, we investigate the frequency of rare *ABCA7* missense mutations, indels, noncanonical splice mutations and compound heterozygous mutations in the Belgian cohort. We analyzed the effect of rare missense mutations in *ABCA7* on the subcellular localization of the protein and observed that mutated ABCA7, transiently expressed in HeLa cells, displays a decreased plasma membrane localization and instead is retained in the endoplasmic reticulum (ER), leading to a loss of functional ABCA7.

## Materials and methods

### Belgian AD patient and control cohorts

Sequencing data of *ABCA7* exons and splice sites of the Belgian AD and control cohort was available from a previous screening effort to investigate the frequency of *ABCA7* PTC mutations [9]. Details about in- and exclusion criteria, demographic and patient characteristics were previously described [9]. In short, the patient cohort consists of 1376 individuals (60.0% [826/1376] women) with a mean age at onset (AAO) of 69.3 ± 10.6 years (range 31–96). Genetic screening of *APP*, *PSEN1* and *PSEN2* was performed in all AD patients and revealed two pathogenic APP mutations (0.15%) and nine pathogenic PSEN1 mutations (0.65%). The control cohort consists of 976 individuals (63.5% [620/976] women) with a mean age at inclusion (AAI) of 72.4 ± 8.6 years (range 43–100).

### Whole exome sequencing

Library preparation and target enrichment was performed using the SeqCap EZ Exome Library v3.0 kit (Nimblegen, Roche, Basel, Switzerland) and sequencing was done on an Illumina® NextSeq platform (Illumina®, San Diego, CA, USA). GenomeComb was used for data processing and variant filtering [41].

### Bioinformatics analysis

Read processing, alignment, variant calling, annotation and downstream filtering of resequencing and whole exome sequencing (WES) data was achieved using GenomeComb [41]. Variants located in segmental duplications, repeated sequences or homo-polymer stretches were considered false positives and were excluded from the study. Only variants with a minor allele frequency (MAF) ≤ 1% in the Genome Aggregation database (GnomAD) v2.1.1 [42], the Exome Variant Server (EVS) (NHLBI GO Exome Sequencing Project (ESP), Seattle, WA (URL: http://evs.gs.washington.edu/EVS/) and the 1000 Genomes Project [43] were retained.

### Sanger sequencing

Validation of all rare (MAF ≤ 1%) non-synonymous coding and splice mutations, identified in the Belgian AD and control cohort’ are performed using Sanger sequencing technology (BigDye Terminator Cycle Sequencing kit v3.1; analysis on an ABI 3730 DNA Analyzer, Thermo Fisher Scientific, MA, US). Sequences were analyzed using NovoSNP [44].

### Haplotype sharing analysis

Haplotypes are based on 18 single nucleotide polymorphisms (SNPs) spanning *ABCA7* and seven short tandem repeat (STR) markers flanking *ABCA7* as described [10]. STR markers were PCR-amplified using fluorescently labelled primers. PCR-products were supplemented with GeneScan™ 500 LIZ™ size standard (Thermo Fisher Scientific, MA, USA) and size-separated using capillary electrophoresis on an ABI 3730 DNA Analyzer (Thermo Fisher Scientific, MA, USA). STR fragment lengths were assigned using the Local Genotype Viewer software (https://www.neuromicssupportfacility.be/) developed by the Neuromics Support Facility, Center for Molecular Neurology, Antwerp, Belgium.

### Preparation of cDNA constructs

The coding sequence of WT human *ABCA7* was purchased in a Gateway®-adapted entry vector from genomics-online (ABIN3417638). The stop codon was removed from the WT-ABCA7 entry vector with in vitro mutagenesis using KAPA HiFi HotStart DNA polymerase (Kapa Biosystems, Wilmington, MA). Afterwards, ten *ABCA7* missense mutations of interest and four likely benign or protective variants were individually introduced in the WT-ABCA7-NoStop entry vector with in vitro mutagenesis. Sequence verified mutant or WT entry clones were subcloned into the in-house developed Gateway®-compatible pCR3 vector with a C-terminal EmGFP-tag to allow the expression of ABCA7-EmGFP fusion proteins. Expression clones were sequence verified. In total, 15 different constructs were generated. Primers used for in vitro mutagenesis are listed in Additional file 1: Table S1.

### Cell culture and transfections

Human cervical carcinoma (HeLa) cells were cultured in Modified Eagle’s medium (MEM; Life Technologies), supplemented with 10% fetal calf serum (Sigma Aldrich), 1% penicillin/streptomycin and 1% L-glutamine (Life Technologies) at 37 °C in a humidified 5% CO_2_ atmosphere.

HeLa cells were transfected using Lipofectamine® 2000 Reagent (Thermofisher) according to the manufacturer’s protocol. Briefly, cells were seeded in a 6-well plate at 2.5 × 10^5^ cells per well, 24 h before transfection. On the day of transfection, cell medium was replaced by medium without antibiotics (Opti-MEM, Life-Technologies). 6 µl Lipofectamine® 2000 Reagent was diluted in 150 µl Opti-MEM and in parallel 2 µg plasmid DNA was added to Opti-MEM in a final volume of 150 µl. The diluted Lipofectamine® 2000 Reagent was added to the diluted DNA and mixed gently by pipetting. After 5 min of incubation at room temperature, the solution was added to the cells in a dropwise manner. 5–6 h after transfection, Opti-MEM was replaced by the original medium.

### Immunocytochemistry and microscopy sample preparation

HeLa cells were reseeded 24 h post-transfection on 12 mm glass coverslips (Fisher Scientific). The next day, cells are fixed for 20 min with 4% EM-grade paraformaldehyde (PFA; Electron Microscopy Sciences 15710) and 4% sucrose in PBS at room temperature and washed three times in PBS.

To visualize the plasma membrane, cells were incubated with wheat germ agglutinin (WGA) conjugated with Alexa Fluor 594 (1:200, Invitrogen w11262) for 10 min at room temperature and washed twice with PBS. Afterwards, the nuclei were stained with Hoechst 33342 (1:2,000; Invitrogen H3570) for 10 min at room temperature and washed twice with PBS. Coverslips were mounted using fluorescent mounting medium (Dako S302380) and stored at 4 °C before being subjected to fluorescence microscopy.

To visualize the ER, fixed cells were permeabilized with 0.5% Triton-X in PBS for 5 min. After washing with PBS, coverslips were blocked with 3% BSA in PBS for 1 h at room temperature. Next, cells were incubated for 2 h with a primary chicken anti-calreticulin antibody as a marker for the ER (1:500, Ab14234, Abcam, USA), followed by three washes with PBS and staining with an Alexa Fluor 594® conjugated goat anti-chicken IgY secondary antibody (1:500, A-11042, Thermo Fisher Scientific, USA) for 1 h. Nuclear staining and mounting were performed as described above.

### Image acquisition and analysis

Confocal images are acquired on a Zeiss LSM700 confocal microscope with Zen 2009 software (Carl Zeiss, Zaventem, Belgium), using an EC Plan-Neofluar 40×/1.30 oil objective. Images of ABCA7-EmGFP and plasma membrane or ER co-markers were acquired in different tracks (serial frame scanning) to avoid any possible crosstalk between the channels. Acquisition settings (laser intensities, detector gain and channel settings) of the individual fluorescence channels were kept identical for the different images. For each genotype and co-marker combination (ABCA7-EmGFP with either WGA or Calreticulin), at least 20 images were taken containing random EmGFP positive cells, resulting in more than 700 multi-channel images in total.

Custom ImageJ scripts [45] were used in Fiji [46] to quantify the colocalization of ABCA7-EmGFP with the plasma membrane and the ER. First, individual cells were delineated using automatic segmentation based on intensity thresholding (Huang method) in the green channel, after noise reduction filtering (Median radius 4) and followed by ROI-size filtering (Analyze particles command). In a next step, the resulting ROI’s were manually corrected to precisely correspond with individual cells and stored in ImageJ ROI manager files. To quantify EmGFP colocalization with WGA or calreticulin in individual cells, an ImageJ script was used that extracts the Pearson’s correlation coefficient between the two fluorescent channels in batch for all ROI’s calling the ImageJ Colocalization Test plugin (www.imagej.net). A minimum of 30 individual cells per genotype were analyzed for each co-marker (WGA or calreticulin), resulting in a total of 1224 cell measurements. ImageJ scripts are written to process and analyze all images (and all genotypes) in batch. Means of the independent measurements for each genotype were compared to the WT using Welch ANOVA with Games-Howell post-hoc test.

## Results

### Rare variants in *ABCA7* in Belgian AD patients and control individuals

**Fig. 1:**
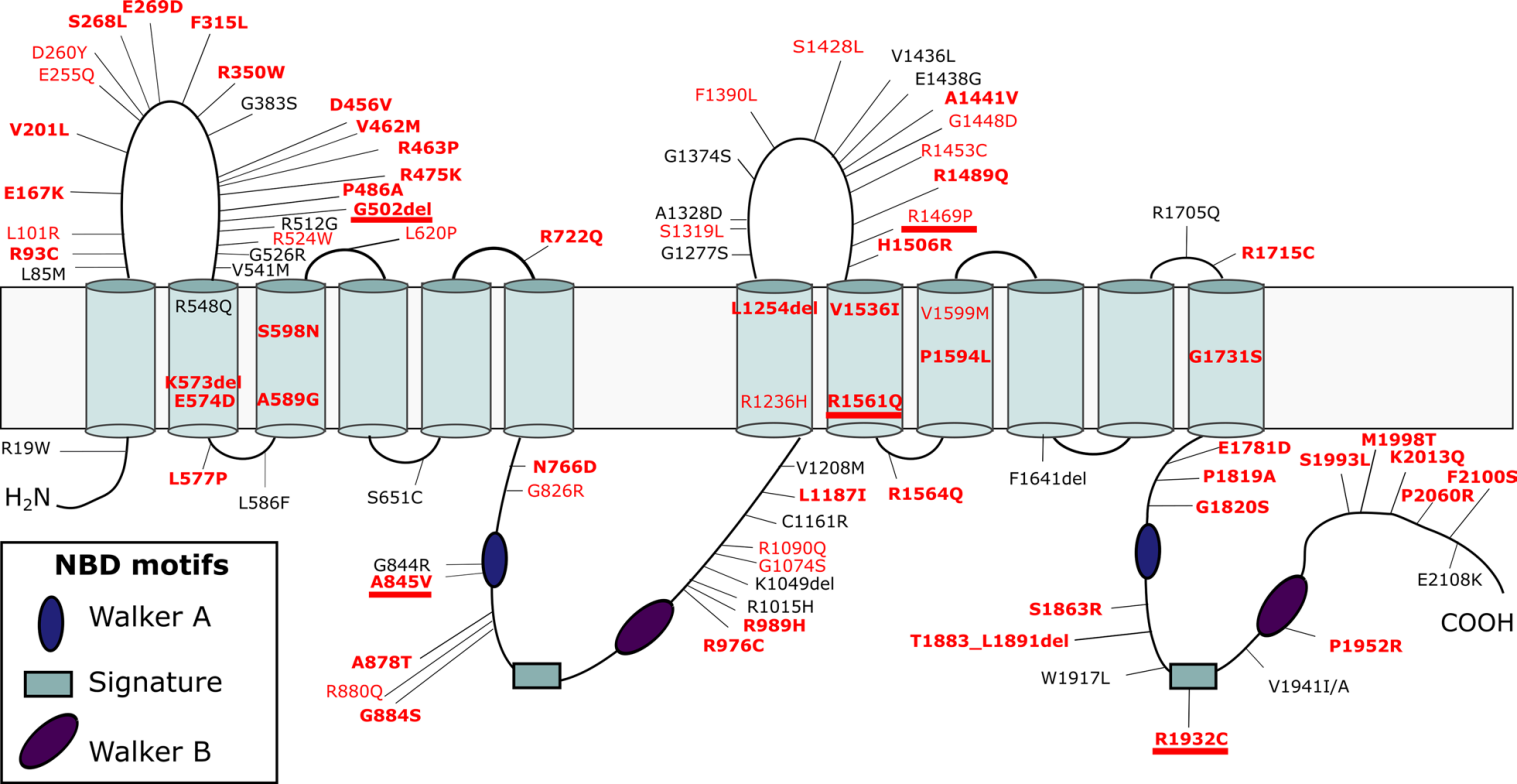
Topological model of ABCA7 with all rare missense mutations and deletions identified in the Belgian cohort. Splice mutations are not indicated in the figure because of their location outside the coding sequence. ABCA7 consists of two transmembrane domains (TMD) with large extracellular loops between the first and second transmembrane helix and two nucleotide binding domains (NBD), containing three motifs. Mutations identified exclusively in control individuals are shown in black. Mutations found in both patients and controls are shown in red. Mutations identified exclusively in patients are indicated in bold. Alignment of the ABCA1, ABCA4 and ABCA7 sequence revealed five mutations (underlined) that correspond to positions in ABCA1 and/or ABCA4 on which disease-associated mutations are found (Additional file 1: Table S6). Protein domain and motif information was based on alignment with ABCA1 [24, 70]

We observed rare (MAF ≤ 1%) variants i.e., missense, indels, and noncanonical splice mutations in the Belgian AD patient cohort (n = 1376) with a frequency of 8.36% (115/1376) and in the Belgian control cohort (n = 976) with a frequency of 6.05% (59/976) (Fig. 1). Carriers of a rare *ABCA7* variant were free from pathogenic mutations in *APP*, *PSEN1* and *PSEN2*, except for one patient carrying both *ABCA7* p.G826R and *PSEN1* p.C263F.

Missense mutations represent the largest group of rare variants in both patients and controls, with some individuals carrying two missense mutations (Additional file 1: Table S2). We identified 101 missense mutations in 96 AD patients (7.34%, 101/1376) and 50 in 46 controls (5.12%, 50/976) (Additional file 1: Table S2). Next, splice mutations are identified in 10 AD patients (0.73%, 10/1376) and seven controls (0.72%, 7/976) (Additional file 1: Table S3). The in silico predicted effect of the identified splice mutations on *ABCA7* mRNA splicing is listed in Additional file 1: Table S4. Indel mutations are found in four AD patients (0.29%, 4/1376) and two controls (0.20%, 2/976) (Additional file 1: Table S5).

Alignment of the ABCA7, ABCA1 and ABCA4 protein sequences indicates that a subset of the rare ABCA7 missense mutations identified in the Belgian cohort affect amino acid residues that are conserved in ABCA1 and/or ABCA4 and correspond to established disease-causing ABCA1 and ABCA4 mutations (Additional file 1: Table S6).

We did not identify homozygous carriers in the Belgian cohort. Nonetheless, we identified 11 patients (0.80%, 11/1376) and five control individuals (0.51%, 5/976) carrying two rare *ABCA7* mutations mutations (*p* = 0.404), and where possible, *cis*/*trans* configuration was determined (Additional file 1: Table S7). Five of the patients carried two missense mutations, four patients combined a missense with a PTC mutation and two patients carried a missense and a splice mutation. Of the control individuals, four carried two missense mutations and one a missense mutation and a PTC mutation (Additional file 1: Table S7). We defined the *cis*/*trans* configuration of the mutations in eight of the 11 patients and in all five controls, confirming *trans* configuration in four patients and in one control (Additional file 1: Table S7). *Cis* configuration was confirmed in another four patients and four controls (Additional file 1: Table S7). In the remaining three AD patients we could not determine *cis*/*trans* configuration. Details of the *cis*/*trans* phasing of the compound heterozygous mutations are available in the Additional file 1: methods and results.

### Co-segregation of p.G1820S with AD in a Belgian family

**Fig. 2:**
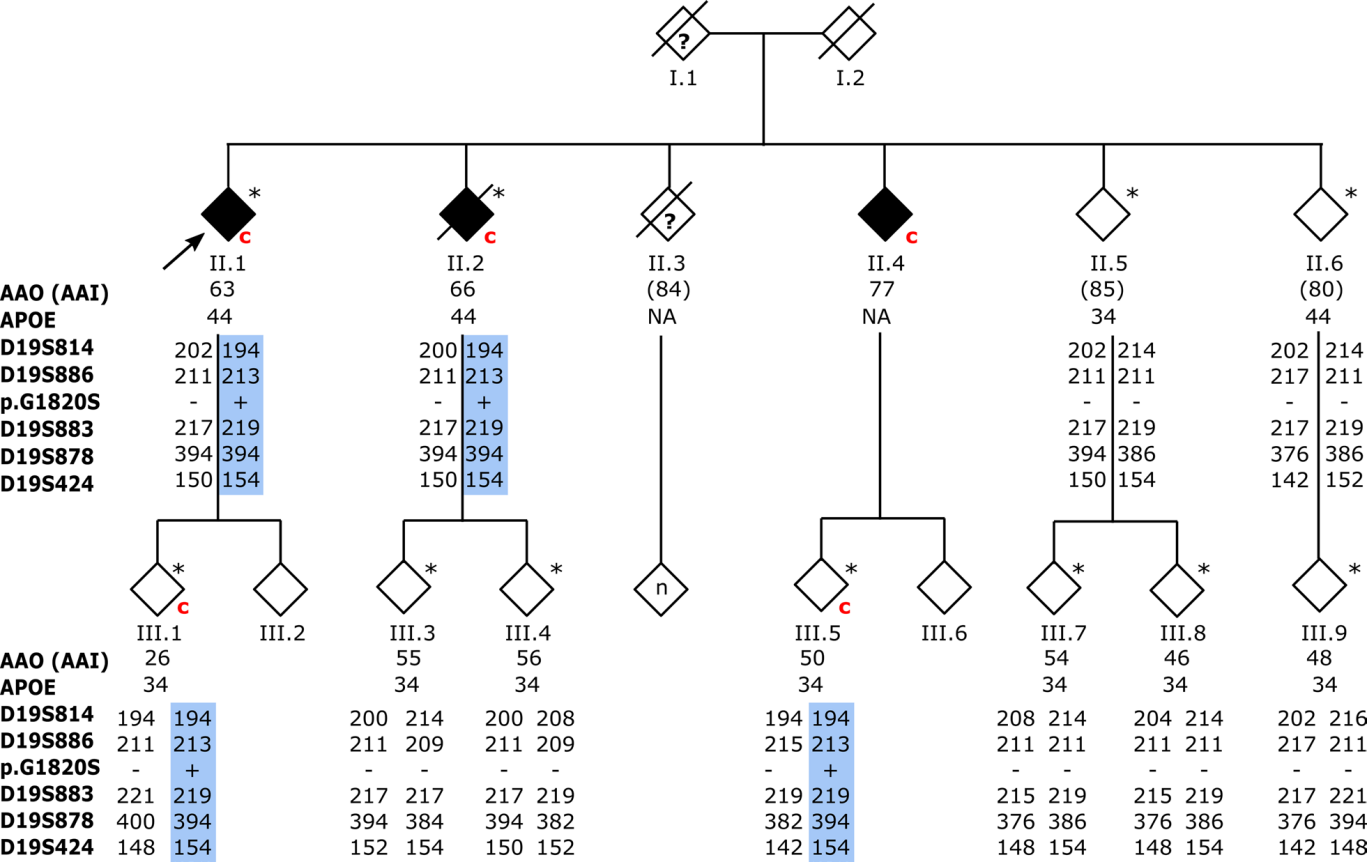
Co-segregation of ABCA7 p.G1820S with AD. To respect the privacy of the participants, the sex of each person is masked, and the pedigree is scrambled. The black arrow indicates the proband. Slashed symbols indicate deceased individuals. Black diamonds indicate individuals diagnosed with AD. Diamonds with question marks represent individuals with hearsay AD. An asterisk represents participants of whom genomic DNA is available. A red ‘c’ represents (obligate) carriers of the *ABCA7* p.G1820S mutation. The shared haplotype of the p.G1820S carriers is indicated in blue. AAO, age at onset; AAI, age at inclusion

For all patient rare variant carriers with a positive familial history, we checked whether DNA of relatives was available for genetic testing. Only one family was informative with several patients and segregation of disease over multiple generations (Fig. 2). The disease pattern in this family mimics autosomal dominant inheritance (Fig. 2). The index patient (II.1) was diagnosed with probable AD at the age of 63 and carries the *ABCA7* p.G1820S mutation. Cerebrospinal fluid analysis shows a pathological biomarker profile compatible with AD. The index patient (II.1) has five siblings of whom two, II.2 and II.4, are diagnosed with probable AD and another sibling, II.3, with AD by hearsay.

In generation II, DNA was available from two affected siblings (II.1 and II.2) and two healthy siblings (II.5 and II.6). Genetic screening disclosed the presence of the *ABCA7* p.G1820S mutation in II.1 and II.2. The II.5 and II.6 siblings tested negative and are cognitively healthy at the advanced ages of 85 and 80 years. In generation III, DNA was available of seven offspring of the three affected and two non-affected individuals of generation II. None of the offspring are affected but III.1 and III.5 are carriers of the *ABCA7* p.G1820S mutation, although they are considerably younger than their affected parents, II.1 and II.4, and therefore possibly presymptomatic. Also, since III.5 is an offspring carrier of II.4, we can consider II.4 an obligate carrier. In addition, whole exome sequencing was performed in affected individuals II.1 and II.2 and unaffected individuals II.5 and II.6 and confirms the absence of co-segregation of other rare variants in Mendelian AD genes and AD-associated genes.

Haplotyping reveals a common haplotype of at least 2.63 Mb shared by all mutation carriers in the family. Within our Belgian AD patient cohort, one additional patient (DR1744.1) with an onset age of 69 years carries the p.G1820S mutation. Haplotyping indicates at least 1.36 Mb sharing with the family demonstrating a genetic relationship due to a distant common ancestor (Additional file 1: Fig. S1).

### *ABCA7* missense mutations affect protein localization in HeLa cells

To investigate whether *ABCA7* missense mutations affect the subcellular localization of ABCA7, immunocytochemistry experiments are performed on HeLa cells transiently transfected with EmGFP-tagged wild type ABCA7 and 10 constructs containing a predicted pathogenic ABCA7 missense mutation. In addition, three constructs containing a likely benign *ABCA7* variant and one construct containing a protective variant are used as positive controls.

The 10 different *ABCA7* missense mutations used in the protein localization experiment are selected from the Belgian cohort (Table 1). We selected mutations with an in silico predicted pathogenic effect, present throughout the full length and the different domains of the protein (Table 1; Fig. 1). In addition, we included mutations that are of particular interest because of their segregation with AD in families (p.R880Q, p.G1820S), because they correspond to Tangier or Stargardt disease mutations in respectively ABCA1 (p.A845V) or ABCA4 (p.R1932C) or because they show enrichment in patients versus controls (p.L620P) (Table 1; Fig. 1). The p.G826R mutation is observed with equal frequency in patients and controls. Of note, all selected mutations affect amino acids that are conserved between ABCA7, ABCA1 and, except for p.G826R, ABCA4. As a positive control, we included in the experiment likely benign variants which are highly frequent in the GnomAD database (p.E188G, p.R1349Q and p.G1527A), and we also included p.G215S, previously shown to have a protective effect against AD in GWAS [38] (Table 1). The minor ‘G’ allele coding for the Glycine at position 1527 is significantly associated with late onset AD in GWAS [5].

**Table 1:** Selected *ABCA7* missense mutations and selected benign variants for subcellular localization studies

cDNA^a^	Protein	PolyPhen-2 (HumDiv)^b^	SIFT^c^	CADD^d^	GnomAD MAF NFE (%)	No. patient carriers [freq. (%)] (n = 1376)	No. control carriers [freq. (%)] (n = 976)
c.1859 T > C	p.L620P	**Probably damaging (1.000)**	**Damaging (0)**	31	0.0555	8 (0.58)	1 (0.10)
c.2476G > A	p.G826R	**Possibly damaging (0.533)**	Tolerated (0.23)	20.7	0.0753	5 (0.37)	4 (0.41)
c.2534C > T	p.A845V	**Probably damaging (1.000)**	**Damaging (0)**	25.2	–	1 (0.07)	–
c.2639G > A	p.R880Q	**Probably damaging (0.999)**	**Damaging (0)**	28.7	0.198	3	1 (0.10)
c.2966G > A	p.R989H	**Probably damaging (1.000)**	**Damaging (0)**	28.2	0.0111	1 (0.07)	–
c.5191G > A	p.G1731S	**Probably damaging (1.000)**	**Damaging (0.01)**	26.5	0.00352	2 (0.15)	–
c.5458G > A	p.G1820S	**Probably damaging (0.994)**	**Damaging (0.03)**	32	0.0462	2 (0.15)	–
c.5794C > T	p.R1932C	**Probably damaging (1.000)**	**Damaging (0)**	26.3	0.00474	1 (0.07)	–
c.5855C > G	p.P1952R	**Probably damaging (1.000)**	**Damaging (0)**	24.9	–	1 (0.07)	–
c.6299 T > C	p.F2100S	**Probably damaging (0.977)**	**Damaging (0)**	26.1	–	1 (0.07)	–
c.563A > G	p.E188G	Benign (0.244)	Tolerated (0.46)	13.13	47.4	NA	NA
c.643G > A	p.G215S	Benign (0.029)	Tolerated (0.92)	0.100	6.34	NA	NA
c.4046G > A	p.R1349Q	Benign (0.002)	Tolerated (0.40)	0.267	43.2	NA	NA
c.4580G > C	p.G1527A	Benign (0.000)	Tolerated (0.89)	3.349	83.0	NA	NA

Bold value represents mutations with a predicted (possible or probable) damaging effect

^a^Coding nomenclature according to NM_019112.3

^b^Polyphen-2 scores [0–1] predict variants as benign (< 0.15), possibly damaging (0.16–0.85) or probably damaging (> 0.85) [65]

^c^SIFT scores range from 0 (damaging) to 1 (tolerated), with a cut-off value of 0.05 [66]

^d^Combined annotation dependent depletion [67]. MAF, minor allele frequency; NFE, non-Finnish Europeans

**Fig. 3:**
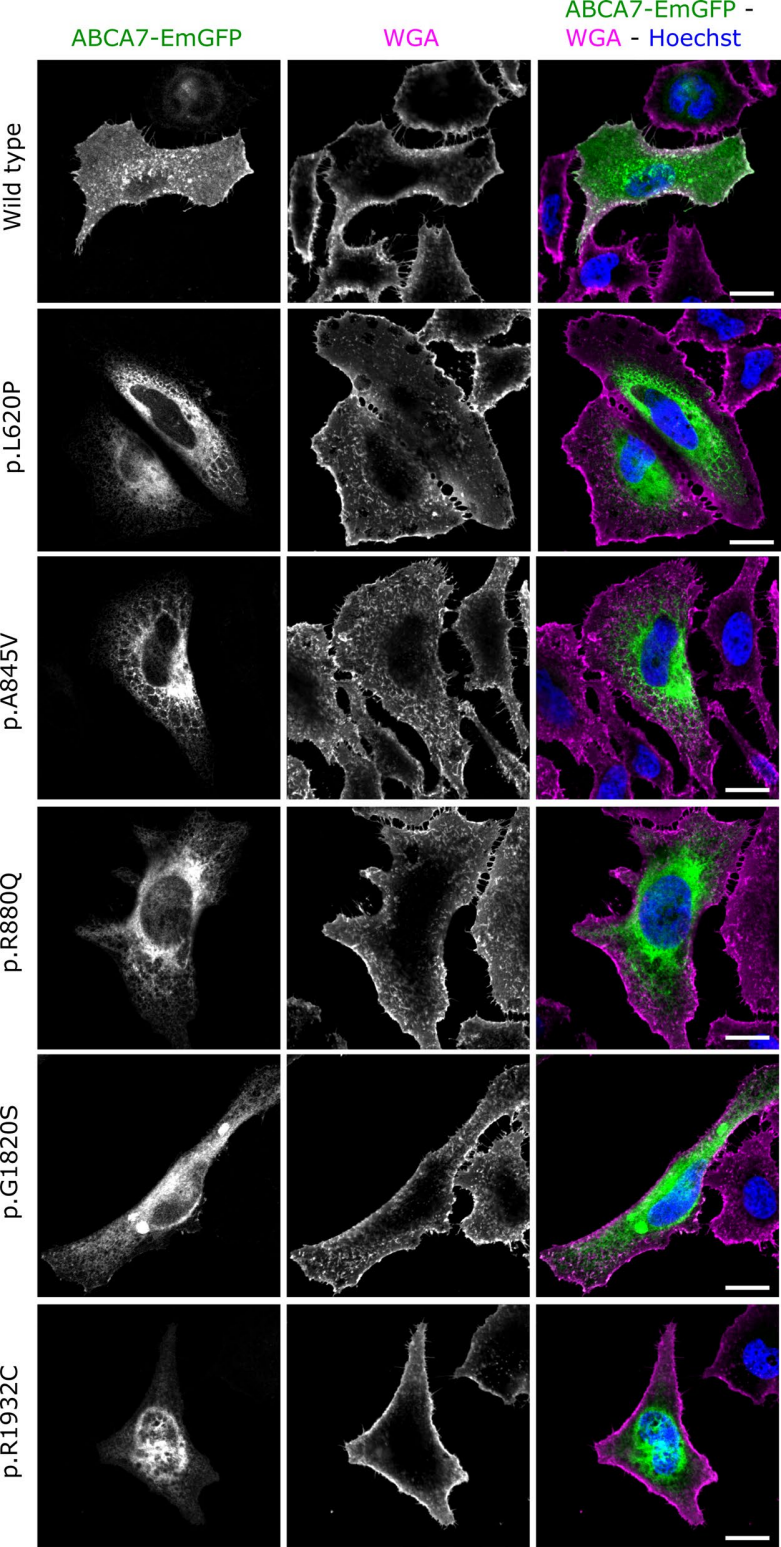
Subcellular localization of WT ABCA7 and mutant ABCA7. Confocal microscopy images of HeLa cells transiently expressing WT or mutant ABCA7-EmGFP. Cells were labelled with WGA (magenta) as a plasma membrane marker. WT ABCA7 locates to the plasma membrane and intracellularly, whereas for the mutants the colocalization with WGA is lost. Instead, the EmGFP signal is concentrated intracellularly and shows a reticulate pattern characteristic for the ER. Scale bars represent 20 µm

**Fig. 4:**
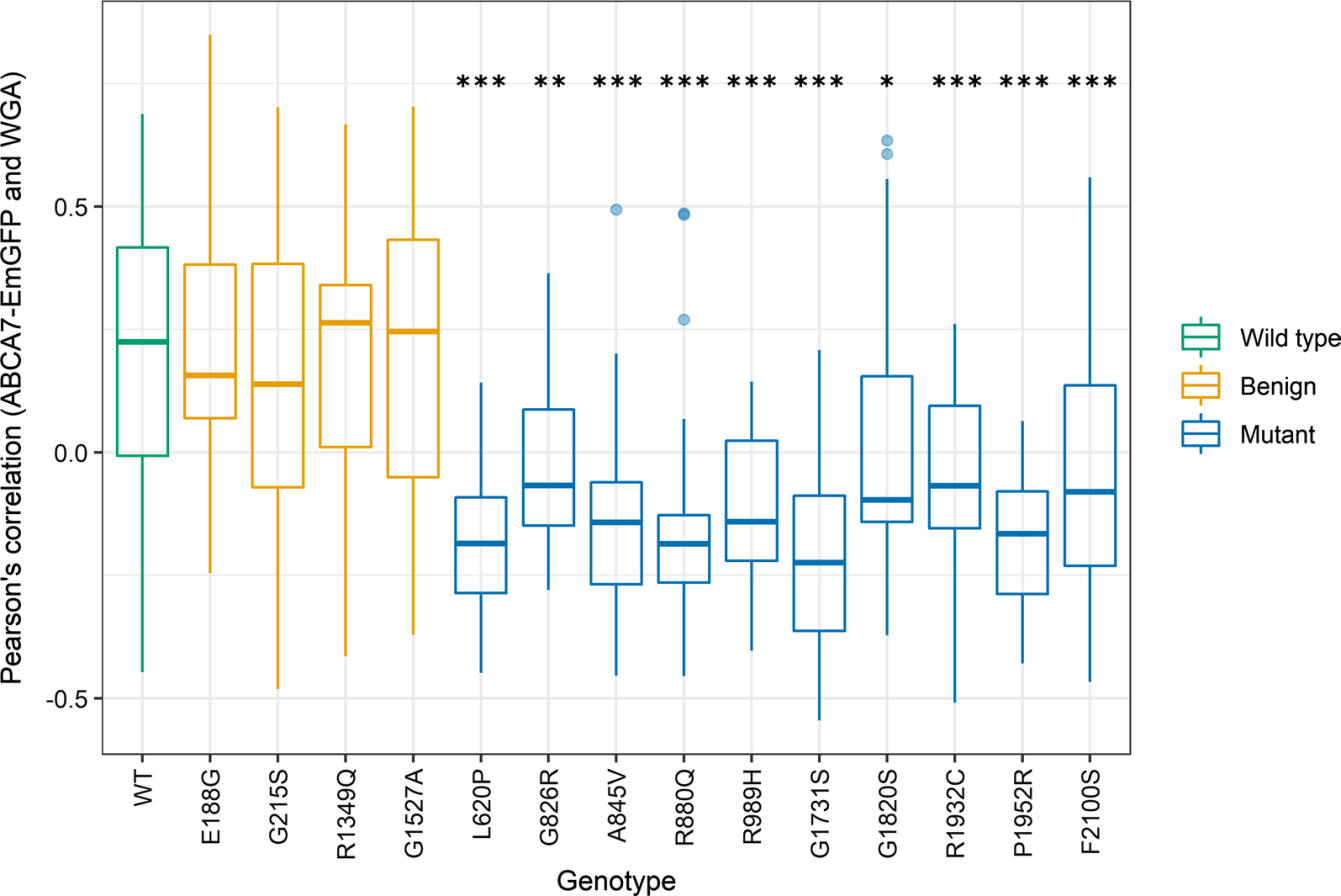
Quantification of the colocalization of ABCA7-EmGFP and the plasma membrane. The Pearson’s correlation coefficient between the ABCA7-EmGFP channel and the WGA channel was measured with ImageJ on a minimum of 30 cells per genotype. Data are represented as median values, with lower and upper quartiles and the value ranges (whiskers). (*p < 0.05; **p < 0.01; ***p < 0.001 after Games-Howell correction)

**Fig. 5:**
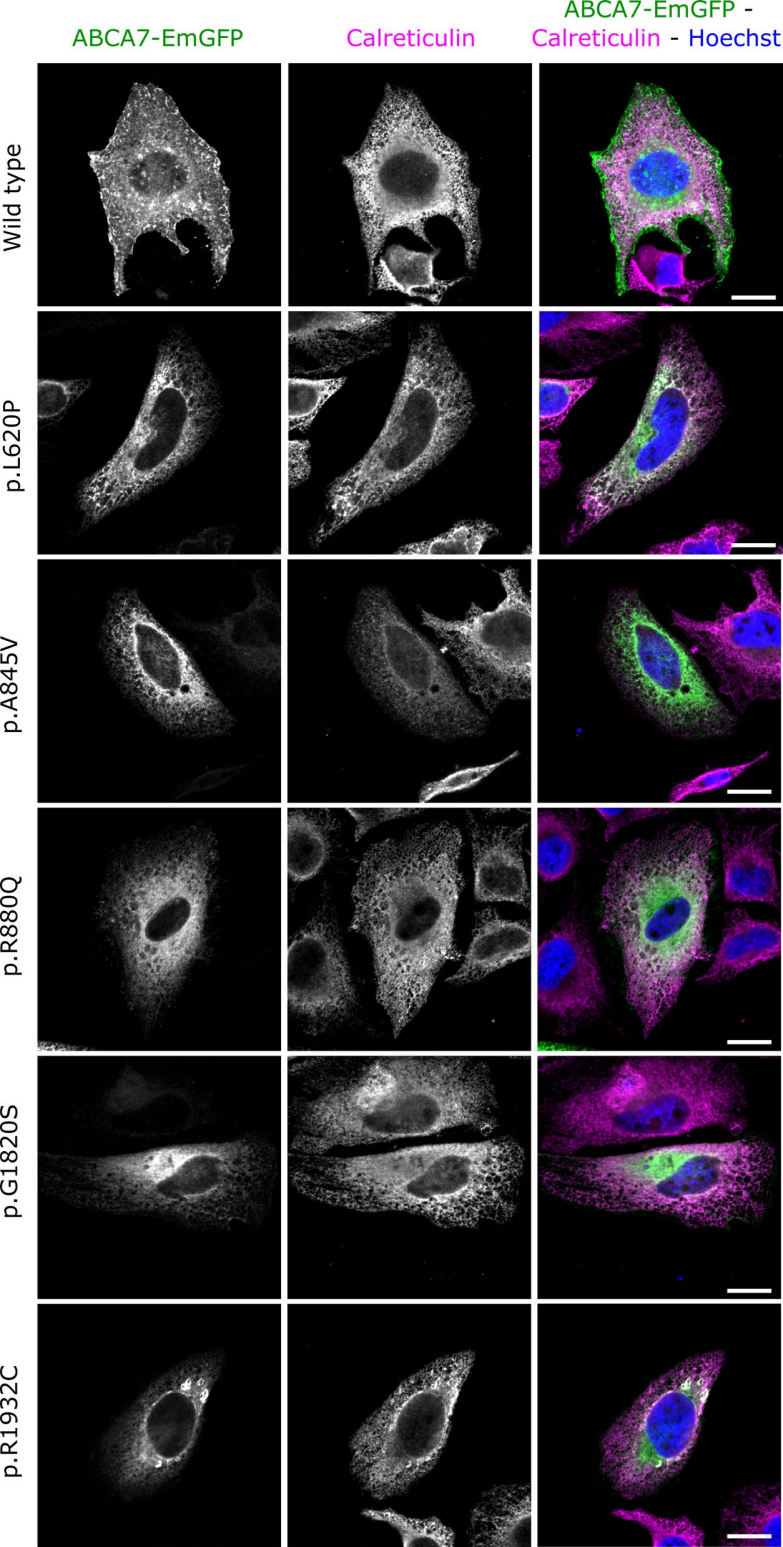
Subcellular localization of WT ABCA7 and mutant ABCA7. Confocal microscopy images of HeLa cells transiently expressing WT or mutant ABCA7-EmGFP. Calreticulin (magenta) was used as a marker for the ER. WT ABCA7 is present inside the ER as well as outside the ER, whereas the expression of mutant ABCA7 is predominantly seen in the ER, as shown by the near perfect colocalization between EmGFP and calreticulin. Scale bars represent 20 µm

**Fig. 6:**
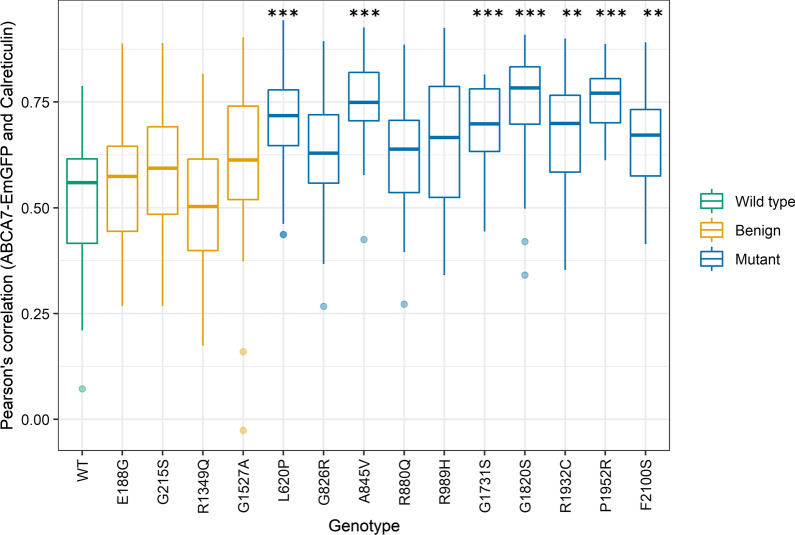
Quantification of the colocalization of ABCA7-EmGFP and the ER. The Pearson’s correlation coefficient between the ABCA7-EmGFP channel and the Calreticulin channel was measured with ImageJ on a minimum of 30 cells per genotype. Data are represented as median values, with lower and upper quartiles and the value ranges (whiskers). (*p < 0.05; **p < 0.01; ***p < 0.001 after Games-Howell correction). Presence of neurofibrillary tangles (arrow) and dystrophic neurites (asterisk) in the hippocampus (**A**). Presence of diffuse plaques and capillary amyloid angiopathy (arrow) in the hippocampus (**B**). Scale bars represent 50 µm. Details on the protocol of the neuropathologic assessment can be found in the Additional file 1: Materials and Method section

The EmGFP signal of cells transfected with WT ABCA7 is present intracellularly with signal accumulations at cell borders, corresponding to the expected location of ABCA7 at the plasma membrane and at intracellular membrane bound organelles e.g., Golgi, endosomes, ER [47]. Co-staining with a marker for the plasma membrane (WGA) demonstrated an obvious overlap with WT ABCA7-EmGFP (Fig. 3). Similarly, HeLa cells transfected with the four ABCA7-EmGFP constructs, containing a likely benign or protective variant (p.E188G, G215S, R1349Q and G1527A), show a similar localization and overlap with the plasma membrane (Additional file 1: Fig. S9). However, hardly any overlap with the plasma membrane is detected when HeLa cells are transfected with any of the mutant ABCA7 constructs (Fig. 3; Additional file 1: Fig. S9). Quantification of the amount of ABCA7 at the plasma membrane, by measuring the Pearson’s correlation coefficient between the WGA and ABCA7-EmGFP fluorescent signals in a high number of cells, confirm the absence of mutant ABCA7 at the plasma membrane (Fig. 4). Instead, mutants generally show an intense intracellular EmGFP signal, in which typical morphological reticular ER-like membrane structures can be distinguished (Fig. 3; Additional file 1: Fig. S9). As expected, co-staining with a marker for the ER (calreticulin) shows a strong colocalization with mutant ABCA7-EmGFP (Fig. 5, Additional file 1: Fig. S10). While WT ABCA7 and benign ABCA7 variants also show some overlap with the ER in addition to their presence on the plasma membrane (Fig. 5; Additional file 1: Fig. S10), quantification by fluorescence colocalization analysis verifies that mutant ABCA7 is retained in the ER (Fig. 6). Confocal microscopy images for the remaining constructs under study are found in the Additional file 1: Data. Overall, the results show a retention of the selected ABCA7 mutants within the ER and indicate aberrant exocytotic processing resulting in an inability to reach the correct cellular destiny at the plasma membrane.

### Clinicopathological phenotype of missense mutation carriers

**Fig. 7:**
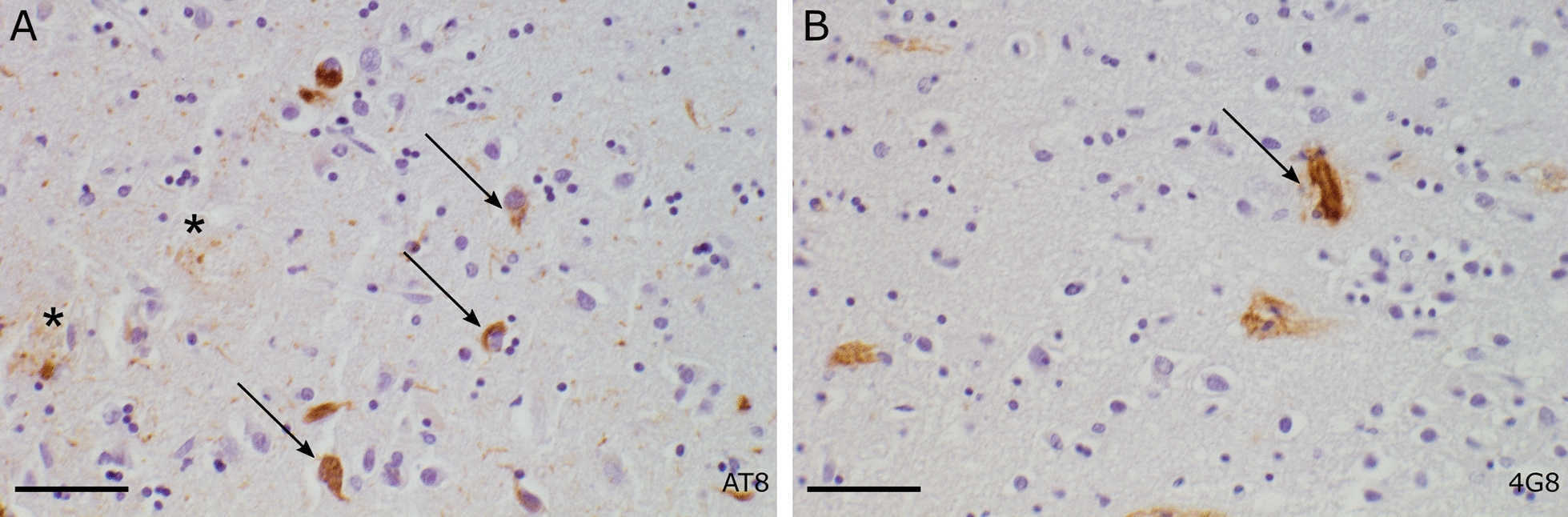
Neuropathological examination of a patient carrying the p.G1731S missense mutation. Presence of neurofibrillary tangles (arrow) and dystrophic neurites (asterisk) in the hippocampus (**A**). Presence of diffuse plaques and capillary amyloid angiopathy (arrow) in the hippocampus (**B**). Scale bars represent 50 µm. Details on the protocol of the neuropathologic assessment can be found in the Additional file 1: Materials and Method section

Mean AAO of missense mutation carriers (n = 96) is 67.9 ± 11.0 years, with a wide range of 37–92 years. Mean age at death (AAD) is 78.3 ± 10.4 years with a mean disease duration (DD) of 8.6 ± 4.3 years. Information on familial history of dementia is available for 73/96 (76.0%) carriers and a positive familial history is noted in 43/73 (58.9%) carriers (Additional file 1: Table S8). The fraction of patients with a missense mutation carrying at least one *APOE* ε4 allele (66/96, 68.8%) is significantly higher compared to the full AD cohort (790/1350, 58.5%) (*p* = 0.002) (Additional file 1: Table S8) [9]. Brain autopsy is performed in six missense mutation carriers (Table 2). High AD neuropathological changes are noted in five carriers, and intermediate changes in one carrier (Table 2, Fig. 7). Meningeal and parenchymal blood vessels present with cerebral amyloid angiopathy (CAA) in all carriers although in different degrees. Capillary CAA (i.e., CAA type 1) is present in all individuals (Table 2).

**Table 2:** Neuropathology of patients with *ABCA7* missense mutations

ID	cDNA^a^	Protein	CADD^b^	AAO/I	AAD	DD	*APOE*	ADNC^c^	CAA^d^	Capillary CAA
1	c.499G > A	p.E167K	15.48	79	89	10	44	A3B3C2	M2P2	+
2	c.1766C > G	p.A589G	0.123	64	75	11	34	A3B3C2	M3P3	+
	c.2476G > A	p.G826R	20.8							
3	c.2632G > A	p.A878T	16.32	51	61	10	33	A3B3C3	M3P3	+
4	c.4357C > T	p.R1453C	23.3	48	57	9	33	A3B3C3	M2P2	+
	c.3148-5C > T	–	0.103							
5	c.4357C > T	p.R1453C	23.3	53	61	8	33	A3B3C3	M2P1-2	+
	c.3148-5C > T	–	0.103							
6	c.5191G > A	p.G1731S	26.4	77	86	9	23	A2B2C1	M1-2P1	+

^a^Coding nomenclature according to NM_019112.3

^b^Combined annotation dependent depletion [67]

^c^Neuropathological changes according to Montine et al. [68]

^d^Scoring for meningeal and parenchymal CAA: 0, no CAA; 1, scant beta amyloid deposition; 2, some circumferential beta amyloid; 3, widespread circumferential beta amyloid, according to Love et al. [69]. AAO/I, age at onset or if not available age at inclusion; AAD, age at death; DD, disease duration; ADNC, Alzheimer's disease neuropathological changes; CAA, cerebral amyloid angiopathy; M, meningeal CAA; P, parenchymal CAA

## Discussion

We investigated the presence of rare (MAF ≤ 1%) *ABCA7* missense, indel and noncanonical splice mutations in the Belgian cohort and identified 115 mutations (8.36%, 115/1376) in the patient cohort In Addition in both patients (7.34%) and controls (5.12%) and are present throughout the full length of the protein, like pathogenic missense mutations in *ABCA1* and *ABCA4* (http://www.hgmd.cf.ac.uk) (Fig. 1). In fact, several of the identified missense mutations in the Belgian AD cohort correspond to pathogenic *ABCA1* or *ABCA4* mutations, indicating a functional role of these amino acids (Additional file 1: Table S6). Taken all rare missense mutations together, in our study we do not observe an enrichment in patients versus controls (SKAT-O *p* = 0.348, Additional file 1: Methods). This might be due to the presence of a high level of benign and protective missense variants. However, the carefully selected mutations included in the immunocytochemistry experiments show an effect on protein localization. Therefore, subgrouping the mutations based on e.g., homology to pathogenic mutations in other ABCA proteins might capture mutations that are likely pathogenic. Nonetheless, functional investigation of single rare missense mutations is required for classification purposes and to estimate their precise contribution to AD.

Missense mutations can affect structure, stability and folding of the protein [48]. Computational modeling can be used to predict the consequence of amino acid changes on protein stability, however, a crystallographic structure of ABCA7 is currently not available. To assess the effect of rare missense mutations on the ABCA7 protein, we investigated the subcellular localization of 10 predicted deleterious missense mutations identified in the Belgian cohort. Previous literature described the presence of a short splicing variant in ABCA7 (type II) in addition to full-length ABCA7 (type I) [47]. In vitro studies with HEK293 cells revealed that both proteins have a different subcellular localization and function [47]. Type I ABCA7 is mainly present at the plasma membrane and at sites corresponding to the intracellular route of exocytosis, while type II ABCA7 is predominantly restricted to the ER and is incapable of ApoA1-mediated lipid release unlike type I ABCA7 [47]. We transiently expressed wild type ABCA7 in HeLa cells and confirm its localization on the plasma membrane and intracellular compartments. We reveal that the 10 mutant ABCA7 constructs under study lead to a significant decrease in ABCA7 plasma membrane expression and are largely retained in the ER, likely impacting their cellular lipid release capacities, while predicted benign variants show the same localization pattern as wild type ABCA7. Our results are in line with data from ABCA1 and ABCA4 studies investigating the effect of missense mutations on protein localization and functional properties. Disease-causing mutations in these genes were found to cause mislocalization and/or to impair the substrate binding or ATPase activity of the transporter [32–34, 36, 49]. Our findings highlight the cellular localization of ABCA7 as a potential therapeutic target for AD, although validation of our results in induced pluripotent stem cell (iPSC) derived brain cells is needed to confirm the pathogenic effect of the studied missense mutations on endogenous ABCA7.

Two of the studied ABCA7 mutants, p.A845V and p.R1932C, are in conserved amino acid residues and mutations at these residues in ABCA1 or ABCA4 are known to cause Tangier disease or Stargardt disease respectively. The p.A845V mutation resides in the Walker A motif of NBD1. The Walker A motif is characterized by the consensus sequence GXNG**A**GKT/S, where X can be any amino acid. This motif is conserved in NBD2 of all members of the ABCA subfamily (ABCA1-13) and in NBD1 of the ABCA subgroup consisting of ABCA1, 2, 3, 4, 7, 12 and 13 [34, 50]. Importantly, the corresponding amino acid substitution of the conserved Alanine to Valine in ABCA1 (p.A937V) is responsible for Tangier disease and highlights the importance of this residue [51]. Interestingly, the ABCA1 p.A937V mutation was found to segregate with AD in a family [52]. In contrast to our results from ABCA7 p.A845V, studies investigating the subcellular location and functional characteristics of ABCA1 p.A937V did not show mislocalization, although cholesterol efflux was abolished, leaving the mutant dysfunctional [53, 54]. p.R1932C is present in the signature motif of NBD2. Substitution of the corresponding basic Arginine to the hydrophobic Tryptophan in ABCA4 (p.R2077W) is identified in a patient with Stargardt disease [32]. Characterization of this variant showed a significantly reduced functional activity as well as mislocalization of ABCA4 indicative of ER-retention [32]. In the ABCA7 p.R1932C, the basic Arginine is replaced for a hydrophobic Cysteine, and we observe a similar pattern of mislocalization.

We also identified patients carrying two rare *ABCA7* alleles, either in *trans* or *cis* configuration. Allen et al. described a remarkably lower brain ABCA7 expression in two c.5570 + 5G > C PTC carriers who caried an additional predicted *ABCA7* deleterious missense mutation compared to two c.5570 + 5G > C patient carriers with normal WT protein expression [7]. A higher mutational burden in *ABCA7* might have a modifying effect on penetrance and pathogenicity. Mutational profiling of *ABCA4* revealed that common variants contribute to increased risk for developing *ABCA4*-related disease and might act as penetrance modifiers when they are combined with a second *ABCA4* mutation [55–57]. In addition, pathogenic deep-intronic variants in *ABCA4* are found to contribute to the recessive inheritance of Stargardt disease [58, 59]. However, in our study we only considered rare (MAF ≤ 1%) coding and splice variants, while additional common, noncoding, or structural variants might as well contribute to the mutational spectrum.

Familial load of missense mutation carriers (58.9%) was lower compared to PTC mutation carriers (77.3%), but still higher than the overall AD cohort (50.0%) [9]. The difference in familial load between PTC and missense carriers might be explained by the bidirectional effect of missense mutations, which is reflected in the high numbers of missense mutations in control individuals. Highly variable onset ages are noted between missense mutation carriers (range 37–92 years old), highlighting once more the need to determine pathogenicity of individual missense mutations as well as the contribution of other genetic factors, e.g., *APOE* genotype. Since the inclusion age of control carriers (range 54–92) falls within the onset age range of patient carriers within our study, we cannot exclude that control carriers may develop AD later in life. Nonetheless, co-segregation of the mislocalizing p.G1820S mutation with AD on a shared haplotype of at least 2.63 Mb in a Belgian AD family with an autosomal dominant inheritance pattern is strengthening our findings on the potential role of *ABCA7* missense mutations in AD. Since individuals II.1 and II.2 both have the APOE ε4/ε4 genotype, which is associated with a higher risk for AD [60], we cannot exclude that both p.G1820S and the *APOE* genotype contribute to the disease. The p.G1820S mutation affects the second NBD of ABCA7 and is predicted deleterious by Mutation Taster, Polyphen2 and SIFT and has a CADD score of 32. In our previous screening of *ABCA7* in a European cohort of 928 EOAD patients, we observed the p.G1820S mutation in a Swedish AD-patient of 64 years old (*APOE* ε33) while it was absent in 980 control individuals [11]. The p.G1820S mutation is also absent in 478 healthy control individuals age 60 or older without neurodegenerative disease from the healthy exome (HEX) database (https://www.alzforum.org/exomes/hex). One earlier study reported co-segregation of the *ABCA7* missense mutation p.R880Q with AD [40]. p.R880Q affects the first NBD of ABCA7 and has a CADD score of 28.7. This mutation is present in three patients and one control individual (0.22% versus 0.10%) within our Belgian cohorts and was found to affect protein localization. All patient carriers reported a positive familial history of dementia. Segregation analysis was not performed since DNA of relatives was not available.

Neuropathological examination in six carriers revealed the hallmarks of AD i.e., senile plaques and neurofibrillary tangles, in combination with (capillary) CAA in all patients. These findings are like the *ABCA7* PTC carriers, in whom extensive levels of CAA and capillary CAA were noted as well (E. Hendrickx Van de Craen et al., personal communication) [61]. Several studies using in vitro and in vivo models have shown that ABCA7 deficiency may lead to a decreased microglial Aβ clearance and an increased APP processing, therefore exacerbating Aβ accumulation in the brain [62]. ABCA1 also plays a role in the pathogenesis of AD and CAA, as increased Aβ deposition as well as increased levels of CAA and CAA-related microhemorrhages were observed in AD mouse models lacking ABCA1 [63, 64].

In conclusion, we describe the frequency of rare *ABCA7* indel, missense and splice mutations in the Belgian AD and control cohorts and explore the pathogenic nature of missense mutations on protein localization in vitro using immunocytochemistry. Our data support a role for rare *ABCA7* missense mutations in the pathogenesis of AD and propose loss of functional ABCA7 at the plasma membrane due to impaired protein localization as the downstream pathogenic mechanism.

## Supplementary Information

**Additional file 1:** Supplementary material and methods.

## Data Availability

All data relevant to this study are included in the research paper or added to the Additional file 1. The corresponding author will share additional information upon reasonable request.

## Abbreviations

AAD: Age at death
AAI: Age at inclusion
AAO: Age at onset age
ABC: ATP-binding cassette
ABCA: ATP-binding cassette subfamily A
AD: Alzheimer’s disease
CAA: Cerebral amyloid angiopathy
CADD: Combined annotation dependent depletion
DD: Disease duration
ER: Endoplasmic reticulum
MAF: Minor allele frequency
NBD: Nucleotide binding domain
NMD: Nonsense-mediated mRNA decay
PTC: Premature termination codon
SNP: Single nucleotide polymorphism
STR: Short tandem repeat
TMD: Transmembrane domain
VNTR: Variable number of tandem repeats
WES: Whole exome sequencing data
WGA: Wheat germ agglutinin
WT: Wild type
