# Supplementary material for: Rare missense mutations in ABCA7 might increase Alzheimer’s disease risk by plasma membrane exclusion
Source: Acta Neuropathol Commun. 2022 Mar 31;10:43. doi: 10.1186/s40478-022-01346-3 (PMC8973822; doi:10.1186/s40478-022-01346-3)
Supplement: Supplementary file 1 — Additional file 1: Supplementary material and methods. [file 40478_2022_1346_MOESM1_ESM.docx]

# **ADDITIONAL FILE 1**

**Rare missense mutations in *ABCA7* might increase Alzheimer’s disease risk by plasma membrane exclusion.**

Liene Bossaerts^1,2#^, Elisabeth Hendrickx Van de Craen^1,2,3#^, Rita Cacace^1,2^, Bob Asselbergh^2,4^, Christine Van Broeckhoven^1,2*^

^1^Neurodegenerative Brain Diseases, VIB Center for Molecular Neurology, Antwerp, Belgium.

^2^Department of Biomedical Sciences, University of Antwerp, Antwerp, Belgium

^3^Department of Neurology, University Hospital Antwerp, Edegem, Belgium.

^4^Neuromics Support Facility, VIB Center for Molecular Neurology, Antwerp, Belgium.

^#^ Sharing first authors

^*^ Corresponding author:

**Prof. Dr. Christine Van Broeckhoven, PhD DSc**

VIB Center for Molecular Neurology

University of Antwerp - CDE

Universiteitsplein 1, B-2610, Antwerpen, België

Tel +32 3 265 1101, Fax +32 3 265 8410

E-Mail: [christine.vanbroeckhoven@uantwerpen.vib.be](mailto:christine.vanbroeckhoven@uantwerpen.vib.be)

**MATERIALS AND METHODS**

## **In silico prediction of splice mutations identified in the Belgian cohort**

In silico evaluation of splice site mutations was done using four splicing prediction algorithms (SpliceSiteFinder-Like, MaxEntScan, NNSPLICE and GeneSplicer) integrated in Alamut Visual version 2.15.0 (Interactive Biosoftware) (**Table S4**).

## **Allele-specific PCR**

Allele-specific PCR is used to determine *cis*/*trans* configuration of compound heterozygous *ABCA7* mutations that are spaced within the size limit of Sanger sequencing. An allele-specific primer and wild type primer, in combination with a general second primer that is placed to obtain amplicons containing both mutations are designed using primer-BLAST [1] to amplify wild type and mutant allele separately. Amplicons are Sanger sequenced and data are analyzed using NovoSNP [2].

## **Long-read DNA sequencing**

Targeted long-read sequencing on a MinION platform with a Flongle flow cell (Oxford Nanopore Technologies, Oxford, UK) is used to determine the *cis*/*trans* configuration of the compound heterozygous mutations. Primers are designed with primer-BLAST to obtain amplicons spanning both mutations. PCR amplification on genomic DNA is performed using LongAmp® Taq 2X Master Mix (New England BioLabs, Ipswich, MA). End-repair and dA-tailing of DNA fragments are performed using the NEBNext FFPE DNA Repair Mix and NEBNext Ultra II End repair/dA-tailing Module (New England BioLabs, Ipswich, MA, USA). Amplicons are barcoded with the Native Barcoding Expansion (Oxford Nanopore Technologies) and ligated with the NEB Blunt/TA Ligase Master Mix (New England BioLabs). Amplicons are pooled equimolar and ONT adapters are ligated on the barcoded, pooled samples using NEBNext Quick Ligation Reaction Buffer (5X) (New England BioLabs). After every step, samples are purified using AMPure XP beads (Beckman Coulter, Brea, CA, USA). Base calling and barcode de-multiplexing is performed with Guppy (v.3.2.2). Alignment to the reference sequence was performed with minimap2 [3]. Variant calling and haplotyping is performed using longshot (v0.4.0) [4].

**Statistical analysis**

Rare variant analysis for missense mutations was performed using an optimized sequence kernel association test (SKAT-O) provided in the SKAT (v2.0.1) package in R (v4.1.0). Gender and *APOE* were included as covariates. First, a power calculation was performed (target sequence = 6834, causal variant percentage = 40%, protective variant percentage = 10%, Maximal OR = 5) using a logistic test for dichotomous traits within the SKAT framework. Under these conditions, our cohort met the required sample size to achieve 80% power.

## **Neuropathologic assessment**

Available neuropathology data of ABCA7 mutation carriers were reviewed and autopsied brain reexamined. After fixation in 10% buffered formalin, brains were embedded in paraffin. Sections were prepared from the cingulate gyrus, frontal superior gyrus, temporal superior gyrus, hippocampus, area striata, mesencephalon, pons, and cerebellum. Histology was performed using Cresyl violet, hematoxylin & eosin, Bodian, and/or Klüver-Barrera. Immunohistochemical analysis was performed with Ubiquitin (Dako, Glostrup, Denmark), AT8 (Innogenetics, Zwijnaarde, Belgium), 4G8 (Signet, Dedham, MA), TDP-43 (Proteintech Group Inc., Chicago, IL), FUS (Proteintech Group Inc.), and/or P62 (BD Diagnostics, Erembodegem, Belgium). We used the previously published methodology for CAA assessment by Love et al. and Thal et al. [5, 6]. This protocol allows scoring parenchymal and meningeal CAA individually on a 0–3 scale and capillary CAA as present/ absent in designated Brodmann areas from the frontal, temporal, parietal and occipital lobes. AD neuropathologic change score was evaluated and staged according to the NIA-AA 2012 criteria [7].

# **RESULTS**

## **Phasing compound heterozygous mutations identified in the Belgian cohorts**

We confirmed trans configuration in four patients and one control individual. In patient P1 (p.R93C/p.R463P), patient P2 (p.A589G/p.G826R) and control individual C4 (p.R1236H/p.V1941A), trans configuration of the variants was confirmed by long-read DNA sequencing. In patient P4, both variants (p.R1489Q/p.H1506R) are located in exon 33 and their trans configuration could be confirmed based on the presence of the variants on separate sequencing reads obtained via targeted resequencing (**Fig. S2**). Genotyping both variants (p.L620P/p.E709fs) showed the presence of only one variant in the child of patient P7, confirming trans configuration in the parent (**Fig. S3**). Cis configuration was confirmed in four patients and four controls. Allele-specific PCR revealed a cis configuration of the variants (p.R1564Q/p.P1594L) present in patient P5 (**Fig. S4**). Long-read sequencing demonstrated a cis configuration in patient P3 (p.S1428L/p.P2060R) and control C5 (p.F1390L/c.4416+2T>G). The same variant combination of control C5 was seen in patient P8 and was present in cis configuration as well, as P8’s child was found to be negative for both variants (**Fig. S5**). Cis configuration was confirmed by segregation analysis for P10 (c.3148-5C>T/p.R1453C), since P10’s child carried both mutations (**Fig. S6**). Control C3’s (p.R512G/p.R1705Q) child carried both mutations, revealing cis confirmation (**Fig. S7**). Lastly, haplotype sharing analysis of controls C1 and C2 (both carrying p.L101R and p.G1277S) together with three carriers of p.L101R as a single mutation, revealed cis configuration (**Fig. S8**). We were unable to phase mutations in patients P6, P9 and P11.

## **Clinical characteristics of patients carrying two *ABCA7* rare variants**

Mean AAO of compound heterozygous missense mutation carriers (n=5) is 62.8 ± 7.7 years, with a mean AAD of 71.0 ± 5.7 and a mean DD of 10.0 ± 1.4 years. At least one *APOE* ε4 allele is present in 1/5 carriers (20.0%) and a positive family history was present in 3/4 (75.0%) of the carriers (**Table S7**). For all 11 compound heterozygous carriers, mean AAO is 62.5 ± 9.1 years, with a mean AAD of 72.0 ± 11.2 and mean DD of 10.6 ± 2.1 years. 4/11 carriers (36.4%) carry at least one *APOE* ε4 allele and 6/7 (85.7%) had a positive family history (**Table S7**).

# **TABLES**

## **Table** **S1** Primer sequences

| **Allele-specific PCR** | | |
| --- | --- | --- |
| **Name** | **Sequence** |  |
| ABCA7_R1564Q_wtF | TTGAGGAGCGAGTCACCCG |  |
| ABCA7_R1564Q_varF | TTGAGGAGCGAGTCACCCA |  |
| ABCA7_R1564Q_R | GGGCCTCACCCATACAGTAG |  |
| ABCA7_P1594L_wtR | AGCACCACGATGCATGCTG |  |
| ABCA7_P1594L_varR | AGCACCACGATGCATGCTA |  |
| ABCA7_P1594L_F | ATGCACCCTCACCCTACAAC |  |
| **ONT long-read sequencing** | | |
| **Name** | **Sequence** |  |
| ABCA7_ONT_P1_F | GGTGACGAAAGCGTTAAGCC |  |
| ABCA7_ONT_P1_R | CAGGAAGGTTCCCAACAGAGC |  |
| ABCA7_ONT_P2_F | GGCTGGCTGGATCAGGTTC |  |
| ABCA7_ONT_P2_R | GTGATGTGGCCCTGGTAGAAG |  |
| ABCA7_ONT_P3_F | CCGGAACCTGTCTGACTTCC |  |
| ABCA7_ONT_P3_R | ACACATGCACACGGTTTCAC |  |
| ABCA7_ ONT_C4_F | GCTGCGGACACAGATATGGA |  |
| ABCA7_ ONT_C4_R | AGGCCTGGATCACCTTAACC |  |
| ABCA7_ ONT_C5_F | ATTGTCTGCAGGTTCTCGGC |  |
| ABCA7_ ONT_C5_R | GGTAGGACGTGCAGGGTAGG |  |
| ***In vitro* mutagenesis** | | |
| **Name** | **Sequence** |  |
| ABCA7_IVM_NoStop_F | GCCGAGACTGTGCTCGGCCTCATGGGCCCAG |  |
| ABCA7_IVM_NoStop_R | CTGGGCCCATGAGGCCGAGCACAGTCTCGGC |  |
| ABCA7_IVM_E188G_F | CTTGTTGGAGGCCGCTG**G**GGACCTGGCCCAGGAGC |  |
| ABCA7_IVM_E188G_R | GCTCCTGGGCCAGGTCC**C**CAGCGGCCTCCAACAAG |  |
| ABCA7_IVM_G215S_F | CCCCGAGGGACCAGC**A**GCCCCCTGGAGTTGC |  |
| ABCA7_IVM_G215S_R | GCAACTCCAGGGGGC**T**GCTGGTCCCTCGGGG |  |
| ABCA7_IVM_L620P_F | CAAGCTGGGAGACATCC**C**CCCCTACAGCCACCCG |  |
| ABCA7_IVM_L620P_R | CGGGTGGCTGTAGGGG**G**GGATGTCTCCCAGCTTG |  |
| ABCA7_IVM_G826R_F | CAGCCAGCCCTGCGG**A**GGCTCAGCCTGGACTTC |  |
| ABCA7_IVM_G826R_R | GAAGTCCAGGCTGAGCC**T**CCGCAGGGCTGGCTG |  |
| ABCA7_IVM_A845V_F | CTGGGCCACAACGGGG**T**CGGCAAGACCACCACC |  |
| ABCA7_IVM_A845V_R | GGTGGTGGTCTTGCCG**A**CCCCGTTGTGGCCCAG |  |
| ABCA7_IVM_R880Q_F | CAGCATGGCCGCCATCC**A**GCCCCACCTGGGCGTC |  |
| ABCA7_IVM_R880Q_R | GACGCCCAGGTGGGGC**T**GGATGGCGGCCATGCTG |  |
| ABCA7_IVM_R989H_F | GCTCAAATACCGAGAAGGTC**A**CACGCTGATCCTCTCCACCC |  |
| ABCA7_IVM_R989H_R | GGGTGGAGAGGATCAGCGTG**T**GACCTTCTCGGTATTTGAGC |  |
| ABCA7_IVM_R1349Q_F | CCTGCCAGTGTAGCC**A**GCCCGGTGCCCGGCG |  |
| ABCA7_IVM_R1349Q_R | CGCCGGGCACCGGGC**T**GGCTACACTGGCAGG |  |
| ABCA7_IVM_G1527A_F | GAGCAGCTGTCTGAGG**C**TGCACTGATGGCCTCC |  |
| ABCA7_IVM_G1527A_R | GGAGGCCATCAGTGCA**G**CCTCAGACAGCTGCTC |  |
| ABCA7_IVM_G1731S_F | CTGCGCTGGGAGGTGGTC**A**GCAAGAACCTCTTGGC |  |
| ABCA7_IVM_G1731S_R | GCCAAGAGGTTCTTGC**T**GACCACCTCCCAGCGCAG |  |
| ABCA7_IVM_R1932C_F | CAGCGGAGGGAACAAA**T**GCAAGCTGGCGACGGC |  |
| ABCA7_IVM_R1932C_R | GCCGTCGCCAGCTTGC**A**TTTGTTCCCTCCGCTG |  |
| ABCA7_IVM_P1952R_F | CGTGGTGTTTCTGGACGAGC**G**GACCACAGGCATGGACC |  |
| ABCA7_IVM_P1952R_R | GGTCCATGCCTGTGGTC**C**GCTCGTCCAGAAACACCACG |  |
| ABCA7_IVM_G1820S_F | CCTGGGGATTCCCCCT**A**GTGAGTGTTTTGGGC |  |
| ABCA7_IVM_G1820S_R | GCCCAAAACACTCAC**T**AGGGGGAATCCCCAGG |  |
| ABCA7_IVM_F2100S_F | GGAGGAGGTATTCTTGTACT**C**CTCCAAGGACCAGGGGAAGG |  |
| ABCA7_IVM_F2100S_R | CCTTCCCCTGGTCCTTGGAG**G**AGTACAAGAATACCTCCTCC |  |

## **Table S2** Rare (MAF ≤1%) *ABCA7* missense mutations identified in the Belgian AD patient and control cohort

| **cDNA^a^** | **Protein** | **No. patient carriers [freq. (%)] (n=1376)** | **No. control carriers [freq. (%)] (n=976)** |
| --- | --- | --- | --- |
| c.55C>T | p.R19W | - | 1 (0.10) |
| c.253C>A | p.L85M | - | 1 (0.10) |
| c.277C>T | p.R93C | 2 (0.15) | - |
| c.302T>G | p.L101R | 2 (0.15) | 3 (0.31) |
| c.499G>A | p.E167K | 1 (0.07) | - |
| c.601G>T | p.V201L | 1 (0.07) | - |
| c.763G>C | p.E255Q | 2 (0.15) | 1 (0.10) |
| c.778G>T | p.D260Y | 2 (0.15) | 1 (0.10) |
| c.803C>T | p.S268L | 1 (0.07) | - |
| c.807G>T | p.E269D | 1 (0.07) | - |
| c.945C>G | p.F315L | 1 (0.07) | - |
| c.1048C>T | p.R350W | 1 (0.07) | - |
| c.1147G>A | p.G383S | - | 1 (0.10) |
| c.1367A>T | p.D456V | 1 (0.07) | - |
| c.1384G>A | p.V462M | 1 (0.07) | - |
| c.1388G>C | p.R463P | 1 (0.07) | - |
| c.1424G>A | p.R475K | 1 (0.07) | - |
| c.1456C>G | p.P486A | 1 (0.07) | - |
| c.1534C>G | p.R512G | - | 1 (0.10) |
| c.1570C>T | p.R524W | 2 (0.15) | - |
| c.1576G>C | p.G526R | - | 1 (0.10) |
| c.1621G>A | p.V541M | - | 1 (0.10) |
| c.1643G>A | p.R548Q | - | 1 (0.10) |
| c.1722G>C | p.E574D | 1 (0.07) | - |
| c.1730T>C | p.L577P | 1 (0.07) | - |
| c.1756C>T | p.L586F | - | 1 (0.10) |
| c.1766C>G | p.A589G | 2 (0.15) | - |
| c.1793G>A | p.S598N | 1 (0.07) | - |
| c.1859T>C | p.L620P | 8 (0.58) | 1 (0.10) |
| c.1952C>G | p.S651C | - | 1 (0.10) |
| c.2165G>A | p.R722Q | 1 (0.07) |  |
| c.2296A>G | p.N766D | 1 (0.07) | - |
| c.2476G>A | p.G826R | 5 (0.37) | 4 (0.41) |
| c.2530G>C | p.G844R | - | 1 (0.10) |
| c.2534C>T | p.A845V | 1 (0.07) | - |
| c.2632G>A | p.A878T | 1 (0.07) | - |
| c.2639G>A | p.R880Q | 3 (0.22) | 1 (0.10) |
| c.2650G>A | p.G884S | 1 (0.07) | - |
| c.2926C>T | p.R976C | 1 (0.07) | - |
| c.2966G>A | p.R989H | 1 (0.07) | - |
| c.3044G>A | p.R1015H | - | 1 (0.10) |
| c.3220G>A | p.G1074S | 1 (0.07) | 1 (0.10) |
| c.3269G>A | p.R1090Q | 1 (0.07) | - |
| c.3481T>C | p.C1161R | - | 1 (0.10) |
| c.3532C>A | p.L1178I | 1 (0.07) | - |
| c.3622G>A | p.V1208M | - | 1 (0.10) |
| c.3707G>A | p.R1236H | 1 (0.07) | 1 (0.10) |
| c.3829G>A | p.G1277S | - | 2 (0.20) |
| c.3956C>T | p.S1319L | 1 (0.07) | 1 (0.10) |
| c.3983C>A | p.A1328D | - | 1 (0.10) |
| c.4120G>A | p.G1374S | - | 1 (0.10) |

## **Table S2** Rare (MAF ≤1%) *ABCA7* missense mutations identified in the Belgian AD patient and control cohort (continued)

|  |  |  |  |
| --- | --- | --- | --- |
| c.4168T>C | p.F1390L | 1 (0.07) | 1 (0.10) |
| c.4283C>T | p.S1428L | 1 (0.07) | 1 (0.10) |
| c.4306G>C | p.V1436L | - | 1 (0.10) |
| c.4313A>G | p.E1438G | - | 1 (0.10) |
| c.4322C>T | p.A1441V | 1 (0.07) | - |
| c.4343G>A | p.G1448D | 5 (0.36) | 1 (0.10) |
| c.4357C>T | p.R1453C | 4 (0.29) | 3 (0.31) |
| c.4466G>A | p.R1489Q | 2 (0.15) | - |
| c.4487G>C | p.R1496P | 1 (0.07) | 1 (0.10) |
| c.4517A>G | p.H1506R | 3 (0.22) | - |
| c.4606G>A | p.V1536I | 1 (0.07) | - |
| c.4682G>A | p.R1561Q | 1 (0.07) | - |
| c.4691G>A | p.R1564Q | 1 (0.07) | - |
| c.4781C>T | p.P1594L | 1 (0.07) | - |
| c.4795G>A | p.V1599M | 9 (0.65) | 5 (0.51) |
| c.5114G>A | p.R1705Q | - | 1 (0.10) |
| c.5143C>T | p.R1715C | 1 (0.07) | - |
| c.5191G>A | p.G1731S | 2 (0.15) | - |
| c.5343G>T | p.E1781D | 1 (0.07) | - |
| c.5455C>G | p.P1819A | 2 (0.15) | - |
| c.5458G>A | p.G1820S | 2 (0.15) | - |
| c.5587A>C | p.S1863R | 1 (0.07) | - |
| c.5750G>T | p.W1917L | - | 1 (0.10) |
| c.5794C>T | p.R1932C | 1 (0.07) | - |
| c.5822T>C | p.V1941I | - | 1 (0.10) |
| c.5822T>C | p.V1941A | - | 1 (0.10) |
| c.5855C>G | p.P1952R | 1 (0.07) | - |
| c.5978C>T | p.S1993L | 1 (0.07) | - |
| c.5993T>C | p.M1998T | 1 (0.07) | - |
| c.6037A>C | p.K2013Q | 1 (0.07) | - |
| c.6179C>G | p.P2060R | 1 (0.07) | - |
| c.6299T>C | p.F2100S | 1 (0.07) | - |
| c.6322G>A | p.E2108K | - | 1 (0.10) |
| **Total** | | **101 (7.3)** | **50 (5.1)** |

^a^Coding nomenclature is according to NM_019112.3.

## **Table S3** Rare (MAF ≤1%) *ABCA7* splice mutations identified in the Belgian AD patient and control cohort

| **cDNA^a^** | **No. patient carriers [freq. (%)]** | **No. control carriers [freq. (%)]** |
| --- | --- | --- |
| c.1622+5G>A | - | 1 (0.10) |
| c.2684+5G>A | 1 (0.07) | - |
| c.2825-7C>T | 4 (0.29) | 1 (0.10) |
| c.3148-5C>T | 2 (0.15) | - |
| c.3727-6C>T | 2 (0.15) | 5 (0.51) |
| c.4206-6C>T | 1 (0.10) | - |
| **Total** | **10 (0.73)** | **7 (0.72)** |

^a^Coding nomenclature is according to NM_019112.3.

## **Table S4** *In silico* predictions of the effect of *ABCA7* splice mutations on mRNA splicing

| **Splicing prediction method**  **([Range], Threshold)** | **c.1622+5G>A** | | **c.2684+5G>A** | | **c.2825-7C>T** | | **c.3148-5C>T** | | **c.3727-6C>T** | | **c.4206-6C>T** | |
| --- | --- | --- | --- | --- | --- | --- | --- | --- | --- | --- | --- | --- |
|  | **Ref.** | **Mutant** | **Ref.** | **Mutant** | **Ref.** | **Mutant** | **Ref.** | **Mutant** | **Ref.** | **Mutant** | **Ref.** | **Mutant** |
| SSF [0-100], ≥ 70 |  |  | 72.24 | 0  (-100%) | 83.88 | 85.39  (+1.8%) | 85.85 | 89.39  (+4.1%) | 73.47 | 75.57  (+2.9%) | 79.62 | 81.73  (+2.6%) |
| MaxEnt [0-12 (16)], ≥ 0 | 4.00 | 0  (-100%) | 5.10 | 0  (-100%) | 10.11 | 10.02  (-0.8%) | 11.76 | 10.38  (-11.7%) | 6.40 | 5.67  (-11.4%) | 10.46 | 9.93  (-5.1%) |
| NNSPLICE [0-1], ≥ 0.4 |  |  | 0.45 | 0  (-100%) | 0.95 | 0.96  (+1.5%) | 0.97 | 0.95  (-1.5%) |  |  | 0.90 | 0.92  (+2.3%) |
| GeneSplicer [0-24 (21)], ≥ 0 | 6.59 | 1.62  (-75.4%) | 7.31 | 0.78  (-89.4%) | 6.58 | 6.45  (-2.0%) | 9.06 | 8.61  (-5.0%) | 1.20 | 0 (-100%) | 10.97 | 9.62  (-12.3%) |

Predicted effect on splicing due to *ABCA7* splice mutations identified in the Belgian cohort, according to four different splicing prediction tools integrated in Alamut Visual version v.2.15.0 (Interactive Biosoftware, Rouen, France): SpliceSiteFinder-like (SSF), MaxEntScan (MaxEnt), NNSPLICE and GeneSplicer. Coding nomenclature is according to NM_019112.3. Splicing scores resulting from the reference and mutated sequences are listed within the complete range of possible values for each method. The difference between the reference sequence score (Ref.) and the mutated sequence score are given in percentage.

## **Table S5** Rare (MAF ≤1%) deletions in *ABCA7* identified in the Belgian AD patient and control cohort

| **cDNA^a^** | **Protein** | **No. patient carriers (freq., %)** | **No. control carriers (freq., %)** |
| --- | --- | --- | --- |
| c.1504_1506del | p.G502del | 1 (0.07) | - |
| c.1717_1719del | p.K573del | 1 (0.07) | - |
| c.3145_3147del | p.K1049del | - | 1 (0.10) |
| c.3760_3762del | p.L1254del | 1 (0.07) | - |
| c.4922_4924del | p.F1641del | - | 1 (0.10) |
| c.5646_5672del | p.T1883_L1891del | 1 (0.07) | - |
| **Total** | | **4 (0.29)** | **2 (0.20)** |

^a^Coding nomenclature is according to NM_019112.3.

## **Table S6** Rare (MAF ≤1%) ABCA7 mutations identified in the Belgian AD cohort and corresponding disease-causing ABCA1 and ABCA4 mutations

| **ABCA7** | **ABCA1** | **ABCA4** | **Functional consequence on ABCA1/ABCA4** |
| --- | --- | --- | --- |
| p.G502del | p.G592D [8] | - | Abolished cholesterol efflux to ApoA1,  significantly reduced cholesterol efflux to HDL particles. |
| p.A845V | p.A937V [9, 10] | - | Impaired ApoA1 mediated cholesterol efflux. |
| p.R1496P | - | p.R1640W [11] | Reduced protein expression on the membrane + reduced ATP-binding capacity. |
| p.R1561Q | - | p.R1705L [12] | Protein misfolding and mislocalization, reduced basal and substrate (i.e., N-Ret-PE) induced ATPase activity, diminished substrate binding and ATP-induced substrate release. |
| p.R1932C | - | p.R2077W [13] | Protein misfolding and mislocalization, drastically reduced basal and substrate induced ATPase activity, significantly reduced substrate binding and ATP-induced substrate release. |

FASTA files with the amino acid sequences of ABCA7, ABCA1 and ABCA4 were downloaded from the UniProt database [14]. Multiple sequence alignment was performed using Clustal Omega [15]. First, all ABCA7 mutations concerning a conserved amino acid in ABCA1 and/or ABCA4 were selected. Afterwards, the Clinvar database [16] and the literature were consulted to check if these ABCA7 mutations correspond to pathogenic ABCA1 and/or ABCA4 mutations. Only ABCA1 and ABCA4 mutants for which functional information is available are included in the list. References are indicated for each mutant.

## **Table S7** Overview and characteristics of AD patients and controls carrying two rare *ABCA7* mutations

| **ID** | **cDNA^a^** | **Protein** | **CADD^b^**  **37v1.6** | ***APOE*** | **AAO** | **AAD** | **DD** | **FH** | ***Cis*/*trans*** | **MAF GnomAD NFE (%)** | **MAF patient cohort (%)** | **MAF control cohort (%)** | **F in patients (%)** | **F in controls (%)** | **F Expected^c^ (%)** |
| --- | --- | --- | --- | --- | --- | --- | --- | --- | --- | --- | --- | --- | --- | --- | --- |
| P1 | c.277C>T  c.1388G>C | p.R93C  p.R463P | 12.81  23.5 | 23 | *62* | *-* | - | F | *Trans* | 0.000963  0.0407 | 0.0727  0.0363 | 0  0 | 0.0727 | 0 | 0.00000904 |
| P2 | c.1766C>G  c.2476G>A | p.A589G  p.G826R | 0.105  20.7 | 34 | *64* | *75* | 11 | F | *Trans* | 0.0290  0.0753 | 0.0727  0.182 | 0  0.205 | 0.0727 | 0 | 0.0000723 |
| P3 | c.4283C>T  c.6179C>G | p.S1428L  p.P2060R | 16.21  22.9 | 33 | *55* | *-* | - | F | *Cis* | 0.0285  0.00236 | 0.0363  0.0363 | 0.0512  0 | 0.0727 | 0 | 0.00000904 |
| P4 | c.4466G>A  c.4517A>G | p.R1489Q  p.H1506R | 23.8  0.001 | 33 | *75* | *-* | - | S | *Trans* | 0.0109  0.00177 | 0.0727  0.109 | 0  0 | 0.0727 | 0 | 0.0000181 |
| P5 | c.4691G>A  c.4781C>T | p.R1564Q  p.P1594L | 17.14  29.9 | 33 | 58 | *67* | 9 | U | *Cis* | 0.00155  - | 0.0363  0.0363 | 0  0 | 0.0727 | 0 | 0.00000452 |
| **All patient double missense mutation carriers (n=5)** | | | | **20.0% *APOE* ε4+** | **62.8±7.7** | **71.0±5.7** | **10.0±1.4** | **75.0%** |  |  |  |  |  |  |  |
| P6 | c.2126_2132del  c.2966G>A | p.E709Afs*85  p.R989H | 32  28.2 | 44 | 74 | 86 | 12 | U | NA | 0.240  0.0111 | 0.581  0.0363 | 0  0 | 0.0727 | 0 | 0.0000723 |
| P7 | c.2126_2132del  c.1859T>C | p.E709Afs*85  p.L620P | 32  31 | 33 | *74* | *85* | 11 | F | *Trans* | 0.240  0.0555 | 0.581  0.291 | 0  0.0512 | 0.0727 | 0 | 0.000651 |
| P8 | c.4416+2T>G  c.4168T>C | -  p.F1390L | 32  24.3 | 34 | *59* | *73* | 14 | U | *Cis* | 0.0381  0.0343 | 0.0363  0.0363 | 0.0512  0.0512 | 0.0727 | 0.102 | 0.0000181 |
| P9 | c.1048C>T  c.5570+5G>C | p.R350W  - | 20.6  22.2 | 34 | 66 | - | - | U | NA | 0.00551  0.378 | 0.0363  0.690 | 0  0.512 | 0.0727 | 0 | 0.000131 |
| P10 | c.3148-5C>T  c.4357C>T | -  p.R1453C | 0.103  23.2 | 33 | *48* | *57* | 9 | F | *Cis* | 0.0341  0.0566 | 0.0727  0.145 | 0  0.154 | 0.145 | 0 | 0.0000633 |
| P11 | c.3148-5C>T  c.4357C>T | -  p.R1453C | 0.103  23.2 | 33 | 53 | 61 | 8 | F | NA | 0.0341  0.0566 | 0.0727  0.145 | 0  0.154 | 0.145 | 0 | 0.0000633 |
| **All patient double mutation carriers (n=11)** | | | | **36.4% *APOE* ε4+** | **62.5±9.1** | **72.0±11.2** | **10.6±2.1** | **85.7%** |  |  |  |  |  |  |  |

**Table S7** Overview and characteristics of AD patients and controls carrying two rare *ABCA7* mutations (continued)

| **ID** | **cDNA^a^** | **Protein** | **CADD**  **37v1.6^b^** | ***APOE*** | **AAI** | ***AAD*** | ***DD*** | ***FH*** | ***Cis*/*trans*** | **MAF GnomAD NFE (%)** | **MAF patient cohort (%)** | **MAF control cohort (%)** | **F in patients (%)** | **F in controls (%)** | **F Expected^c^ (%)** |
| --- | --- | --- | --- | --- | --- | --- | --- | --- | --- | --- | --- | --- | --- | --- | --- |
| C1 | c.302T>G  c.3829G>A | p.L101R  p.G1277S | 25.6  24.1 | 34 | 73 | *-* | *-* | *-* | *Cis* | 0.140  0.00444 | 0.0727  0 | 0.154  0 | 0 | 0.205 | 0.0000452 |
| C2 | c.302T>G  c.3829G>A | p.L101R  p.G1277S | 25.6  24.1 | 33 | 71 | *-* | *-* | *-* | *Cis* | 0.140  0.00444 | 0.0727  0 | 0.154  0 | 0 | 0.205 | 0.0000452 |
| C3 | c.1534C>G  c.5114G>A | p.R512G  p.R1705Q | 14.45  23.2 | 24 | 73 | *-* | *-* | *-* | *Cis* | 0.00807  0.000776 | 0  0 | 0.0512  0.0512 | 0 | 0.102 | 0.00000452 |
| C4 | c.3707G>A  c.5822T>C | p.R1236H  p.V1941A | 22.9  24 | 34 | 87 | *-* | *-* | *-* | *Trans* | 0.00329  0.00896 | 0.0363  0 | 0.0512  0 | 0 | 0.102 | 0.00000904 |
| C5 | c.4416+2T>G  c.4168T>C | -  p.F1390L | 32  24.3 | 33 | 84 | *-* | *-* | *-* | *Cis* | 0.0381  0.0343 | 0.0363  0.0363 | 0.0512  0.0512 | 0.0727 | 0.102 | 0.0000181 |
| **All control double mutation carriers**  **(n = 5)** | | | | **60.0% *APOE* ε4+** | **77.6±7.3** |  |  |  |  |  |  |  |  |  |  |

^a^Coding nomenclature is according to NM_019112.3. ^b^Combined annotation dependent depletion [17]. ^c^Expected frequency is calculated according to the Hardy-Weinberg principle, using the MAF of the single alleles in patients and controls. AAO, age at onset; AAI, age at inclusion; AAD, age at death; DD, disease duration; FH, familial history; CADD, combined annotation dependent depletion; MAF, minor allele frequency; F, frequency of compound heterozygotes.

**Table S8** Characteristics of patients carrying an *ABCA7* missense mutation

| **ID** | **cDNA^a^** | **Protein** | **CADD^b^**  **37v1.6** | ***APOE*** | **AAO** | **AAD** | **DD** | **FH** |
| --- | --- | --- | --- | --- | --- | --- | --- | --- |
| P1 | c.277C>T | p.R93C | 12.81 | 44 | 72 | 77 | 5 | F |
| P2 | c.277C>T  c.1388G>C | p.R93C  p.R463P | 12.81  23.5 | 23 | 62 | - | - | F |
| P3 | c.302T>G | p.L101R | 25.6 | 34 | 63 | (70) | - | S |
| P4 | c.302T>G | p.L101R | 25.6 | 33 | 64 | - | - | F |
| P5 | c.499G>A | p.E167K | 17.75 | 44 | 79 | 89 | 10 | S |
| P6 | c.601G>T | p.V201L | 4.422 | 24 | 59 | (67) | - | S |
| P7 | c.763G>C | p.E255Q | 6.331 | 33 | 76 | 87 | 11 | S |
| P8 | c.763G>C | p.E255Q | 6.331 | 44 | 53 | 57 | 4 | S |
| P9 | c.778G>T | p.D260Y | 22.8 | 34 | 73 | 78 | 5 | U |
| P10 | c.778G>T | p.D260Y | 22.8 | 34 | 60 | - | - | F |
| P11 | c.803C>T | p.S268L | 11.4 | 34 | 73 | - | - | S |
| P12 | c.807G>T | p.E269D | 12.26 | 33 | 69 | - | - | F |
| P13 | c.945C>G | p.F315L | 18.86 | 34 | 79 | 87 | 8 | S |
| P14 | c.1048C>T  c.5570+5G>C | p.R350W  - | 20.6 | 34 | 66 | - | - | U |
| P15 | c.1367A>T | p.D456V | 0.954 | 24 | 40 | - | - | U |
| P16 | c.1384G>A | p.V462M | 18.83 | 34 | 58 | (75) | - | S |
| P17 | c.1424G>A | p.R475K | 15.95 | 34 | 76 | - | - | F |
| P18 | c.1456C>G | p.P486A | 23.1 | 34 | 88 | 94 | 6 | S |
| P19 | c.1570C>T | p.R524W | 22.3 | 33 | 69 | 72 | 3 | S |
| P20 | c.1570C>T | p.R524W | 22.3 | 33 | 56 | - | - | S |
| P21 | c.1722G>C | p.E574D | 24.2 | 33 | 72 | - | - | U |
| P22 | c.1730T>C | p.L577P | 28 | 34 | 75 | - | - | S |
| P23 | c.C1766G  c.G2476A | p.A589G  p.G826R | 0.105  20.7 | 34 | 64 | 75 | 11 | F |
| P24 | c.1766C>G | p.A589G | 0.105 | 34 | 70 | (76) | - | S |
| P25 | c.1793G>A | p.S598N | 20.4 | 34 | 80 | - | - | F |
| P26 | c.1859T>C  c.2126_2132del | p.L620P  p.E709fs | 31 | 33 | 74 | 85 | 11 | F |
| P27 | c.1859T>C | p.L620P | 31 | 33 | 70 | - | - | F |
| P28 | c.1859T>C | p.L620P | 31 | 24 | 85 | - | - | U |
| P29 | c.1859T>C | p.L620P | 31 | 33 | 41 | (59) | - | F |
| P30 | c.1859T>C | p.L620P | 31 | 33 | 48 | (66) | - | F |
| P31 | c.1859T>C | p.L620P | 31 | 23 | 63 | - | - | U |
| P32 | c.1859T>C | p.L620P | 31 | 34 | 67 | 72 | 5 | F |
| P33 | c.1859T>C | p.L620P | 31 | 44 | 56 | - | - | S |
| P34 | c.2165G>A | p.R722Q | 13.97 | 23 | 57 | - | - | F |
| P35 | c.2296A>G | p.N766D | 20.6 | 44 | 71 | - | - | S |
| P36 | c.2476G>A | p.G826R | 20.7 | 34 | 76 | - | - | S |
| P37 | c.2476G>A | p.G826R | 20.7 | 44 | 73 | 80 | 7 | F |
| P38 | c.2476G>A | p.G826R | 20.7 | 34 | 62 | 78 | 16 | U |
| P39* | c.2476G>A | p.G826R | 20.7 | 33 | 60 | - | - | F |
| P40 | c.2534C>T | p.A845V | 25.2 | 33 | <83 | - | - | F |
| P41 | c.2632G>A | p.A878T | 16.32 | 33 | 51 | 61 | 10 | F |
| P42 | c.2639G>A | p.R880Q | 28.7 | 34 | 84 | - | - | F |
| P43 | c.2639G>A | p.R880Q | 28.7 | 34 | 71 | 84 | 13 | F |
| P44 | c.2639G>A | p.R880Q | 28.7 | 34 | 64 | - | - | F |
| P45 | c.2650G>A | p.G884S | 26.7 | 44 | 78 | - | - | U |
| P46 | c.2926C>T | p.R976C | 24.7 | 34 | 69 | (83) | - | F |
| P47 | c.2966G>A  c.2126_2132del | p.R989H  p.E709fs | 28.2 | 44 | 74 | 86 | 12 | U |
| P48 | c.3220G>A | p.G1074S | 29.6 | 34 | 67 | - | - | S |
| P49 | c.3269G>A | p.R1090Q | 9.902 | 34 | 79 | 85 | 6 | S |
| P50 | c.3532C>A | p.L1178I | 5.836 | 33 | 56 | - | - | F |
| P51 | c.3707G>A | p.R1236H | 22.9 | 24 | 67 | (79) | - | S |

## **Table S8** Characteristics of patients carrying an *ABCA7* missense mutation (continued)

| **ID** | **cDNA^a^** | **Protein** | **CADD^b^**  **37v1.6** | ***APOE*** | **AAO** | **AAD** | **DD** | **FH** |
| --- | --- | --- | --- | --- | --- | --- | --- | --- |
| P52 | c.3956C>T | p.S1319L | 2.82 | 33 | 85 | 90 | 5 | F |
| P53 | c.4168T>C  c.4416+2T>G | p.F1390L  - | 24.3 | 34 | 59 | 73 | 14 | U |
| P54 | c.4283C>T  c.6179C>G | p.S1428L  p.P2060R | 16.21  22.9 | 33 | 55 | - | - | F |
| P55 | c.4322C>T | p.A1441V | 0.564 | 44 | 68 | - | - | F |
| P56 | c.4343G>A | p.G1448D | 13.21 | 33 | 57 | 66 | 9 | U |
| P57 | c.4343G>A | p.G1448D | 13.21 | 34 | 73 | (80) | - | F |
| P58 | c.4343G>A | p.G1448D | 13.21 | 34 | 67 | 68 | 1 | F |
| P59 | c.4343G>A | p.G1448D | 13.21 | 44 | 65 | - | - | F |
| P60 | c.4343G>A | p.G1448D | 13.21 | 34 | 52 | - | - | S |
| P61 | c.4357C>T | p.R1453C | 23.2 | 34 | 67 | - | - | F |
| P62 | c.4357C>T  c.3148-5C>T | p.R1453C  - | 23.2 | 33 | 53 | 61 | 8 | F |
| P63 | c.4357C>T | p.R1453C | 23.2 | 34 | 60 | - | - | U |
| P64 | c.4357C>T  c.3148-5C>T | p.R1453C  - | 23.2 | 33 | 48 | 57 | 9 | F |
| P65 | c.4466G>A  c.4517A>G | p.R1489Q  p.H1506R | 23.8  0.001 | 33 | 75 | - | - | S |
| P66 | c.4466G>A | p.R1489Q | 23.8 | 44 | 61 | - | - | S |
| P67 | c.4487G>C | p.R1496P | 15.37 | 34 | 58 | 79 | 21 | S |
| P68 | c.4517A>G | p.H1506R | 0.001 | 34 | 71 | - | - | F |
| P69 | c.4517A>G | p.H1506R | 0.001 | 34 | 74 | 88 | 14 | F |
| P70 | c.4606G>A | p.V1536I | 23.4 | 34 | 92 | - | - | U |
| P71 | c.4682G>A | p.R1561Q | 28.1 | 34 | 64 | - | - | S |
| P72 | c.4691G>A  c.4781C>T | p.R1564Q  p.P1594L | 17.14  29.9 | 33 | 58 | 67 | 9 | U |
| P73 | c.4795G>A | p.V1599M | 25.5 | 34 | 81 | - | - | U |
| P74 | c.4795G>A | p.V1599M | 25.5 | 34 | 86 | - | - | S |
| P75 | c.4795G>A | p.V1599M | 25.5 | 33 | 80 | 83 | 3 | U |
| P76 | c.4795G>A | p.V1599M | 25.5 | 34 | 37 | (50) | - | U |
| P77 | c.4795G>A | p.V1599M | 25.5 | 44 | 62 | - | - | S |
| P78 | c.4795G>A | p.V1599M | 25.5 | 44 | 59 | - | - | S |
| P79 | c.4795G>A | p.V1599M | 25.5 | 33 | 69 | - | - | F |
| P80 | c.4795G>A | p.V1599M | 25.5 | 44 | 71 | - | - | F |
| P81 | c.4795G>A | p.V1599M | 25.5 | 44 | 66 | (71) | - | U |
| P82 | c.5143C>T | p.R1715C | 27.2 | 34 | 66 | - | - | F |
| P83 | c.5191G>A | p.G1731S | 26.5 | 23 | 77 | 86 | 9 | U |
| P84 | c.5191G>A | p.G1731S | 26.5 | 34 | <71 | (84) | - | U |
| P85 | c.5343G>T | p.E1781D | 10.79 | 34 | 77 | - | - | U |
| P86 | c.5455C>G | p.P1819A | 17.12 | 44 | 84 | 89 | 5 | U |
| P87 | c.5455C>G | p.P1819A | 17.12 | 34 | 79 | 85 | 6 | F |
| P88 | c.5458G>A | p.G1820S | 32 | 44 | 68 | - | - | F |
| P89 | c.5458G>A | p.G1820S | 32 | 44 | 63 | (74) | - | F |
| P90 | c.5587A>C | p.S1863R | 7.085 | 34 | 74 | - | - | S |
| P91 | c.5794C>T | p.R1932C | 26.3 | 34 | 81 | - | - | F |
| P92 | c.5855C>G | p.P1952R | 24.9 | 34 | 78 | 87 | 9 | S |
| P93 | c.5978C>T | p.S1993L | 23.7 | 33 | 64 | (72) | - | F |
| P94 | c.5993T>C | p.M1998T | 25 | 33 | 89 | - | - | U |
| P95 | c.6037A>C | p.K2013Q | 24.4 | 33 | 75 | - | - | S |
| P96 | c.6299T>C | p.F2100S | 26.1 | 44 | 61 | - | - | F |
| **All patients (n=96)** | | | | **68.8% *APOE* ε4+** | **67.9±11.0** | **78.3±10.4** | **8.5±4.3** | **58.9%** |

^a^Coding nomenclature is according to NM_019112.3. ^b^Combined annotation dependent depletion [18]. AAO, age at onset; AAD, age at death; DD, disease duration; FH, familial history.

## **Table S9** Characteristics of controls carrying an *ABCA7* missense mutation.

| **ID** | **cDNA^a^** | **Protein** | **CADD^b^**  **37v1.6** | ***APOE*** | **AAI** |
| --- | --- | --- | --- | --- | --- |
| C1 | c.55C>T | p.R19W | 23.9 | 33 | 80 |
| C2 | c.253C>A | p.L85M | 14.01 | 34 | 55 |
| C3 | c.302T>G  c.3829G>A | p.L101R  p.G1277S | 25.6  24.1 | 33 | 72 |
| C4 | c.302T>G | p.L101R | 25.6 | 33 | 92 |
| C5 | c.302T>G  c.3829G>A | p.L101R  p.G1277S | 25.6  24.1 | 34 | 73 |
| C6 | c.763G>C | p.E255Q | 6.331 | 34 | 77 |
| C7 | c.778G>T | p.D260Y | 22.8 | 33 | 76 |
| C8 | c.1147G>A | p.G383S | 16.92 | 34 | 70 |
| C9 | c.1534C>G  c.5114G>A | p.R512G  p.R1705Q | 16.45  23.2 | 24 | 76 |
| C10 | c.1576G>C | p.G526R | 22.4 | 33 | 71 |
| C11 | c.1621G>A | p.V541M | 19.47 | 34 | 70 |
| C12 | c.1643G>A | p.R548Q | 25.5 | 33 | 89 |
| C13 | c.1756C>T | p.L586F | 24.7 | 33 | 71 |
| C14 | c.1859T>C | p.L620P | 31 | 23 | 85 |
| C15 | c.1952C>G | p.S651C | 23.5 | 34 | 76 |
| C16 | c.2476G>A | p.G826R | 20.7 | 33 | 67 |
| C17 | c.2476G>A | p.G826R | 20.7 | 34 | 59 |
| C18 | c.2476G>A | p.G826R | 20.7 | 33 | 74 |
| C19 | c.2476G>A | p.G826R | 20.7 | 23 | 60 |
| C20 | c.2530G>C | p.G844R | 26.2 | 33 | 89 |
| C21 | c.2639G>A | p.R880Q | 28.7 | 33 | 74 |
| C22 | c.3044G>A | p.R1015H | 23.2 | 33 | 65 |
| C23 | c.3220G>A | p.G1074S | 29.6 | 33 | 78 |
| C24 | c.3481T>C | p.C1161R | 2.843 | 34 | 68 |
| C25 | c.3622G>A | p.V1208M | 11.87 | 33 | 83 |
| C26 | c.3707G>A  c.5822T>C | p.R1236H  p.V1941A | 22.9  24 | 34 | 87 |
| C27 | c.3956C>T | p.S1319L | 2.82 | 33 | 66 |
| C28 | c.3983C>A | p.A1328D | 15.13 | 33 | 68 |
| C29 | c.4120G>A | p.G1374S | 0.11 | 33 | 66 |
| C30 | c.4168T>C  c.4416+2T>G | p.F1390L  - | 24.3 | 33 | 84 |
| C31 | c.4283C>T | p.S1428L | 16.21 | 33 | 70 |
| C32 | c.4306G>C | p.V1436L | 0.089 | 34 | 82 |
| C33 | c.4313A>G | p.E1438G | 15.73 | 34 | 72 |
| C34 | c.4343G>A | p.G1448D | 13.21 | 23 | 61 |
| C35 | c.4357C>T | p.R1453C | 23.2 | 33 | 91 |
| C36 | c.4357C>T | p.R1453C | 23.2 | 33 | 54 |
| C37 | c.4357C>T | p.R1453C | 23.2 | 33 | 56 |
| C38 | c.4487G>C | p.R1496P | 15.37 | 33 | 74 |
| C39 | c.4795G>A | p.V1599M | 25.5 | 24 | 64 |
| C40 | c.4795G>A | p.V1599M | 25.5 | 23 | 78 |
| C41 | c.4795G>A | p.V1599M | 25.5 | 23 | 64 |
| C42 | c.4795G>A | p.V1599M | 25.5 | 34 | 77 |
| C43 | c.4795G>A | p.V1599M | 25.5 | 33 | 73 |
| C44 | c.5750G>T | p.W1917L | 9.789 | 33 | 82 |
| C45 | c.5822T>C | p.V1941I | 14.74 | 33 | 80 |
| C46 | c.6322G>A | p.E2108K | 22.3 | 33 | 78 |
| **All controls (n=46)** | | | | **30.4% *APOE* ε4+** | **73.4±9.5** |

^a^Coding nomenclature is according to NM_019112.3. ^b^Combined annotation dependent depletion [17]. AAI, age at inclusion.

**FIGURES**

**
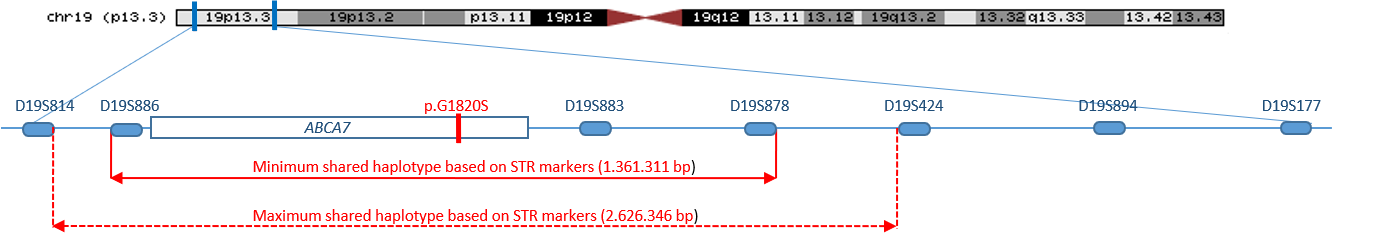
**

**Fig. S1** Genomic map of STR-markers. The minimum shared haplotype (at least 1.36 Mb) of the family with DR1744.1 is indicated with a solid red arrow. The maximum shared haplotype (2.626.346 bp or 2.63 Mb) is indicated with dashed arrows.

**
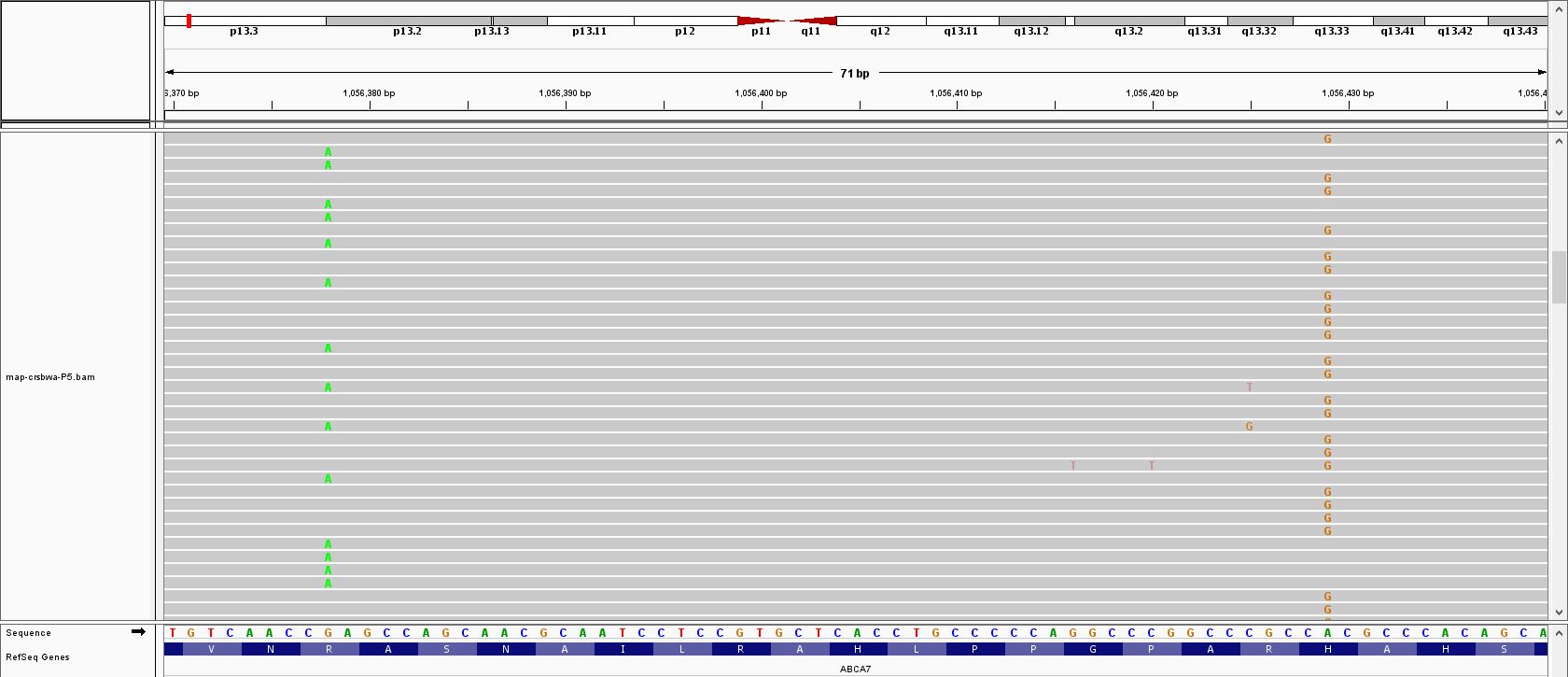
**

**Fig. S2** p.R1489Q and p.H1506R are located in *trans* in patient P5. Visualisation of the .bam file of patient P5 using Integrative Genome Viewer (IGV) [11]. Mutations do not occur on the same sequencing reads, confirming *trans* configuration.

**
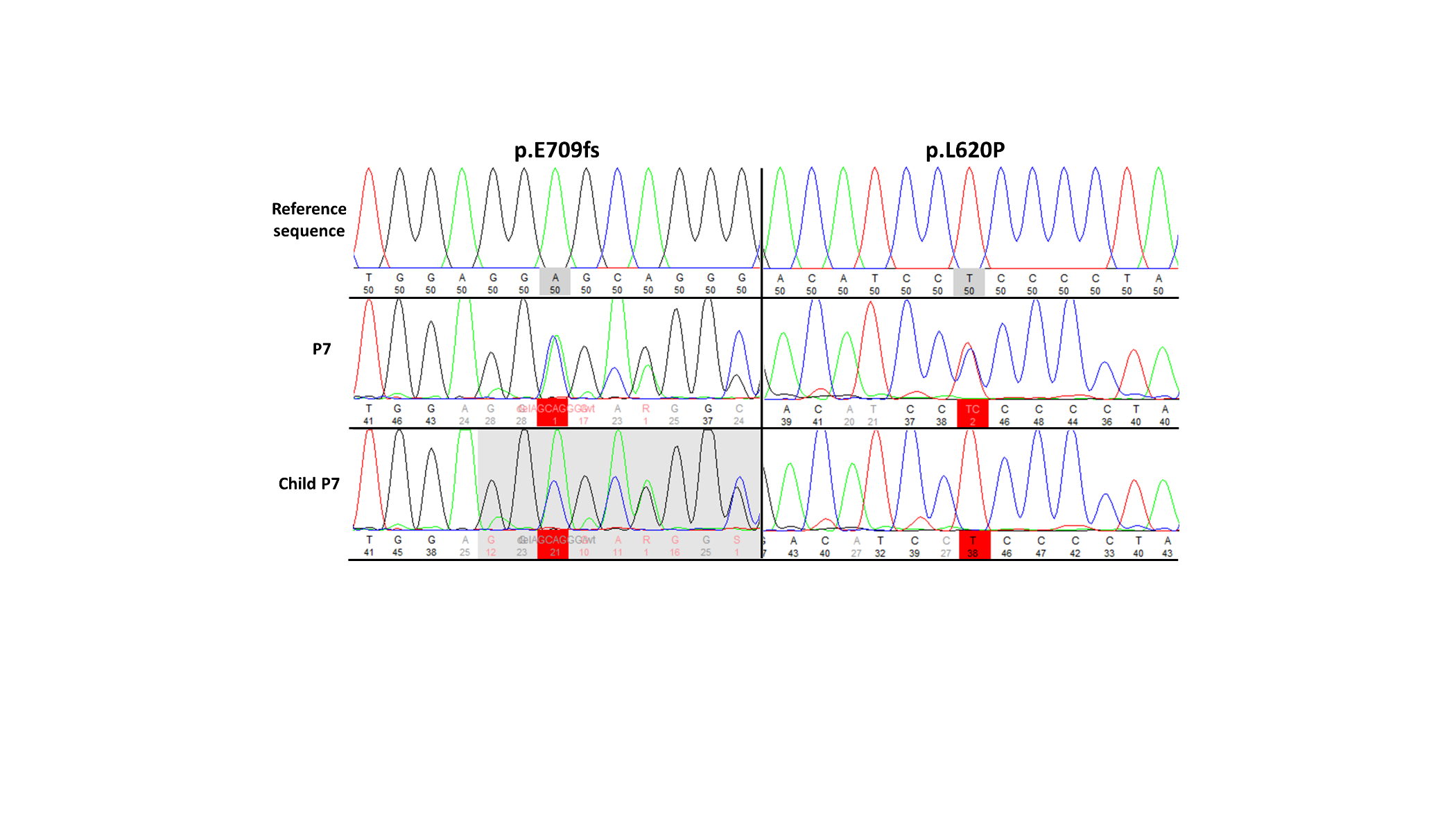
**

**Fig. S3** p.E709fs and p.L620P are located in *trans* in patient P7. Sanger sequencing results show that P7’s child carriers only one mutation (p.E709fs), confirming *trans* configuration of the mutations in the parent (P7).


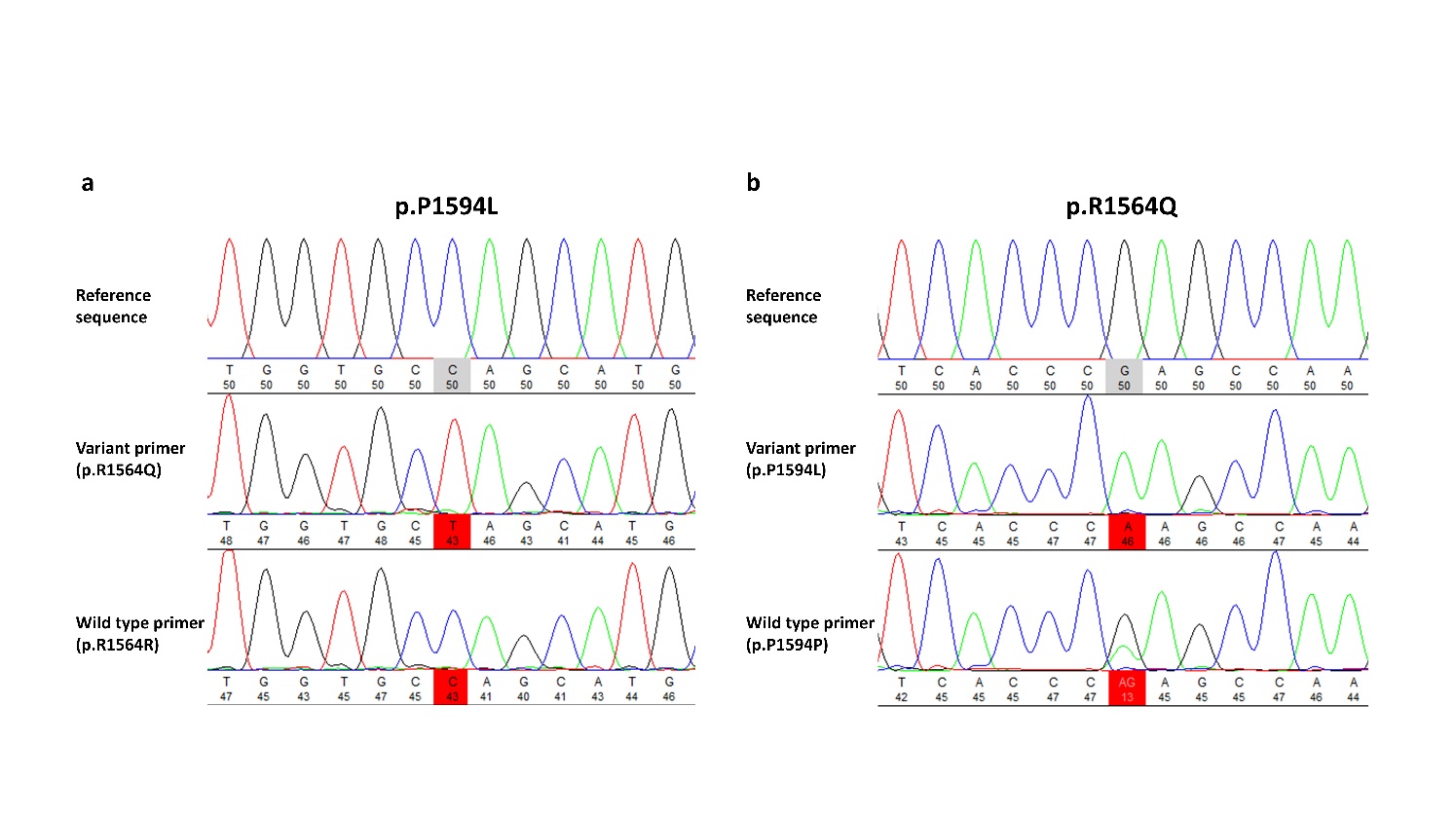


**Fig. S4** Allele-specific PCR shows *cis* configuration of mutations p.R1564Q and p.P1594L in patient P5. **a)** Two different forward primers were designed: one containing the wild type nucleotide and one containing the variant nucleotide of p.R1564Q. When using the p.R1564Q variant primer for PCR amplification, the p.P1594L mutant is present in the Sanger sequencing reads, while amplification using the wild type p.R1564R primer shows reads without the presence of p.P1594L. **b**) Likewise, two different reverse primers were designed: one containing the wild type nucleotide and one containing the variant nucleotide of p.P1594L. Accordingly, PCR amplification using the p.P1594L variant primer resulted in reads containing the p.R1564Q mutant. The p.P1594P wild type primer shows nonspecific binding resulting in reads with and without p.P1594L.


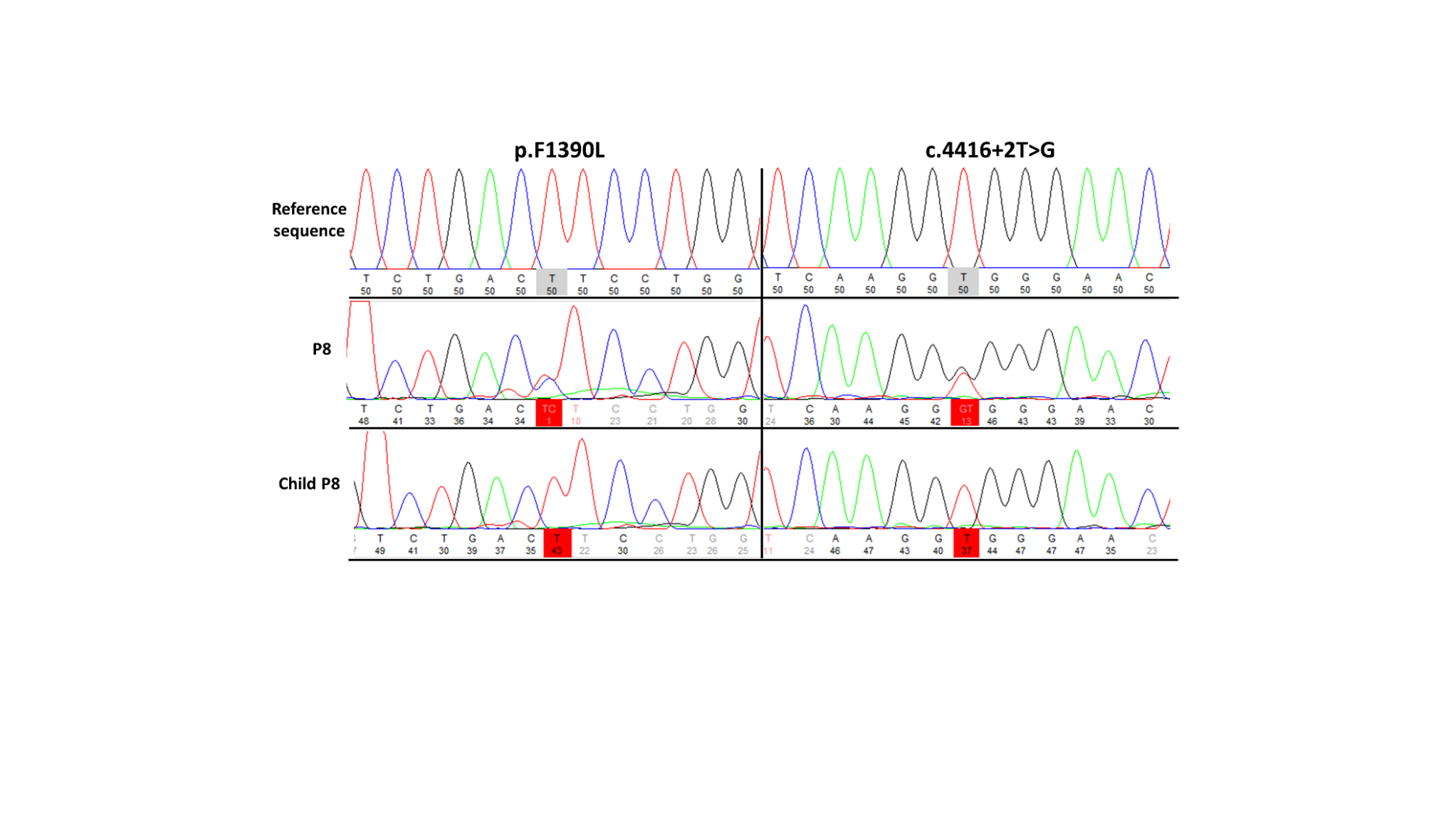


**Fig. S5** p.F1390L and c.4416+2T>G are in *cis* in patient P8. Sanger sequencing results show that both mutations of patient P8 are absent in P8’s child, confirming *cis* configuration of the mutations.


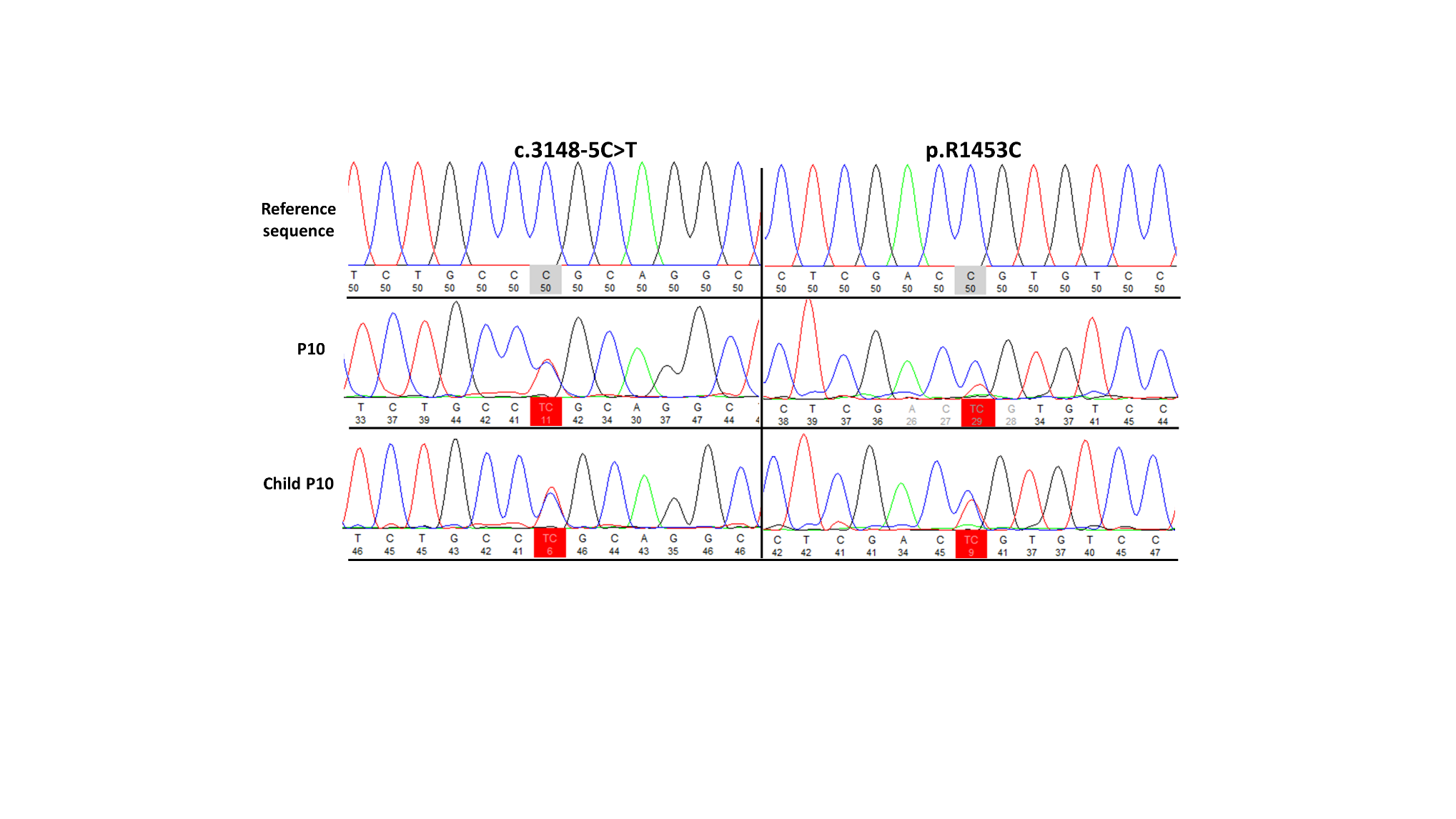


**Fig. S6** c.3148-5C>T and p.R1453C are located in *cis* in patient P10. Sanger sequencing results show that P10’s child carries both mutations, confirming *cis* configuration of the mutations.


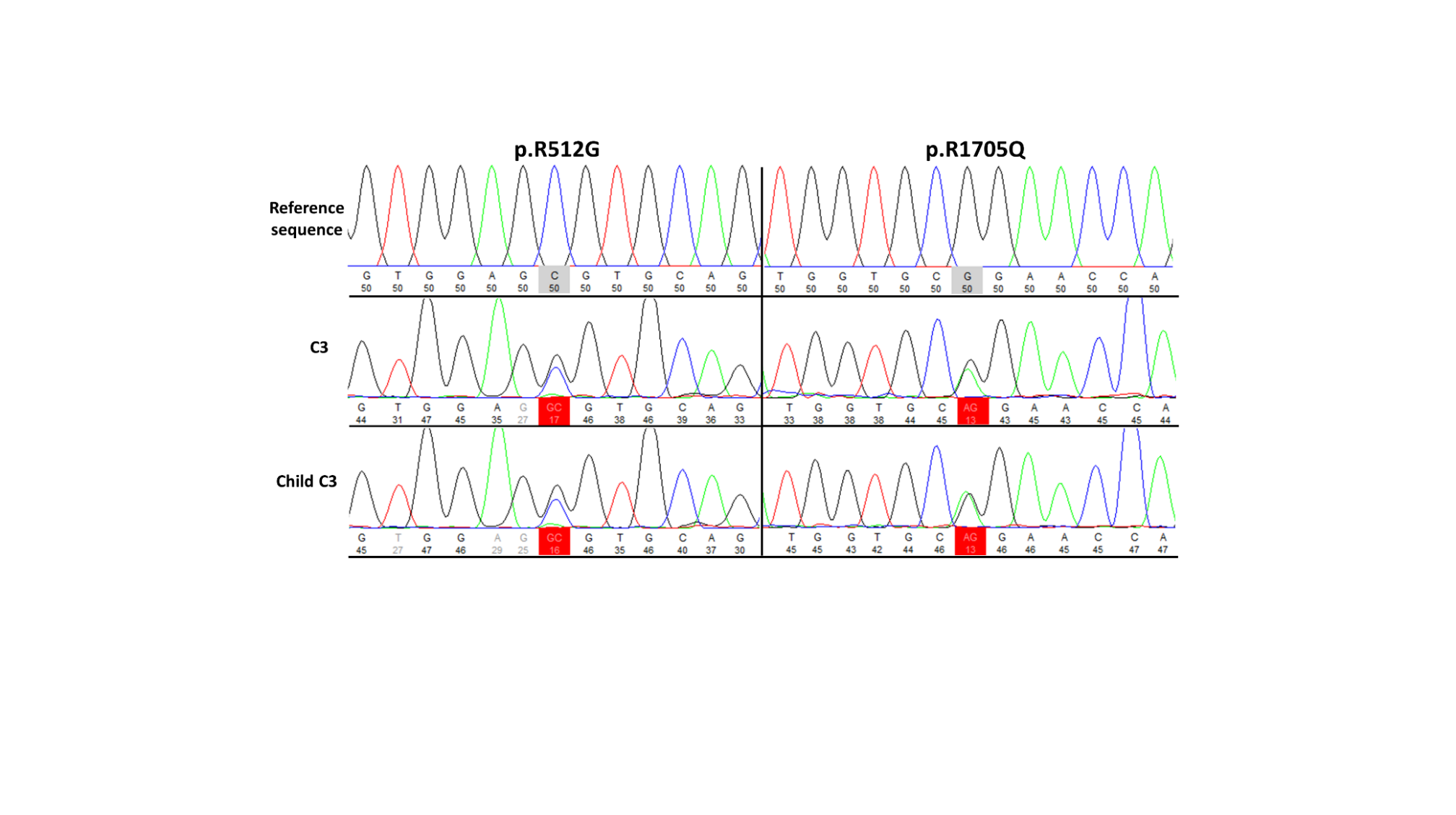


**Fig. S7** p.R512G and p.R1705Q are in *cis* configuration in control individual C3. Sanger sequencing results show that C3’s child carries both mutations, confirming *cis* configuration of the mutations.


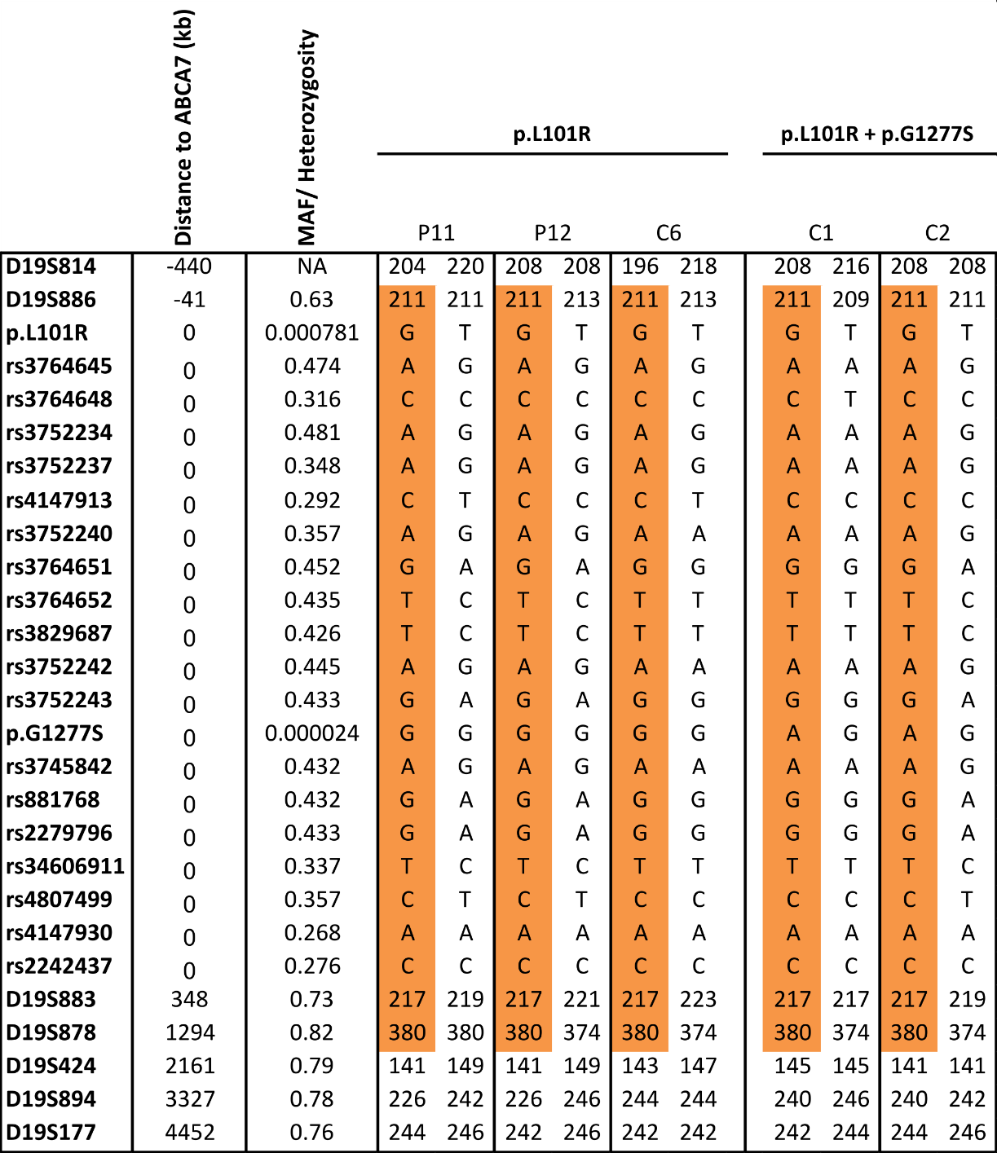


**Fig. S8** Haplotype sharing analysis of p.L101R carriers and p.L101R + p.G1277S carriers reveals *cis* configuration of the mutations. Heterozygosity’s of STR markers are obtained from the Marshfield Clinic database (www.marshfieldclinic.org). Minor allele frequencies (MAF) of SNPs are obtained from the GnomAD (non-Finnish European) database [12].

**
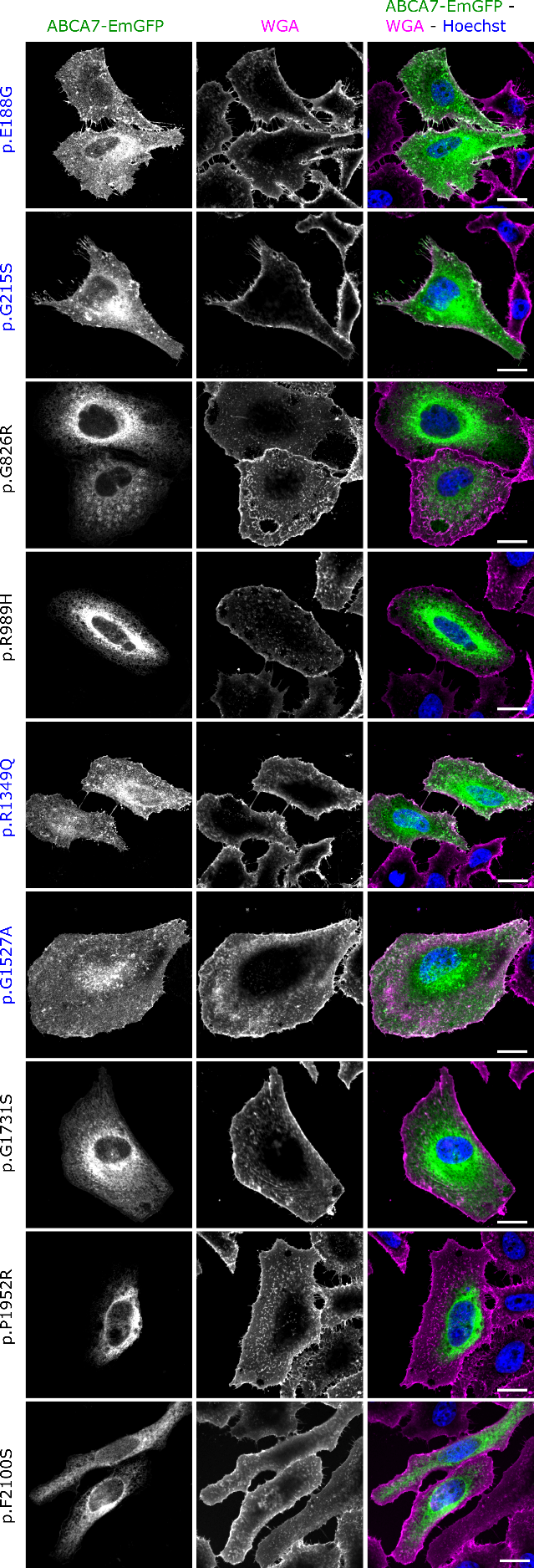
**

**Fig. S9** Subcellular localization of individual mutant and benign ABCA7-EmGFP constructs. Confocal microscopy images of HeLa cells transiently expressing ABCA7-EmGFP are shown. Predicted pathogenic mutants are indicated with black text color, predicted benign variants are indicated with blue text color. Cells were labelled with WGA (magenta) as a plasma membrane marker to investigate colocalization between ABCA7 and the plasma membrane. Scale bars represent 20µm.

**
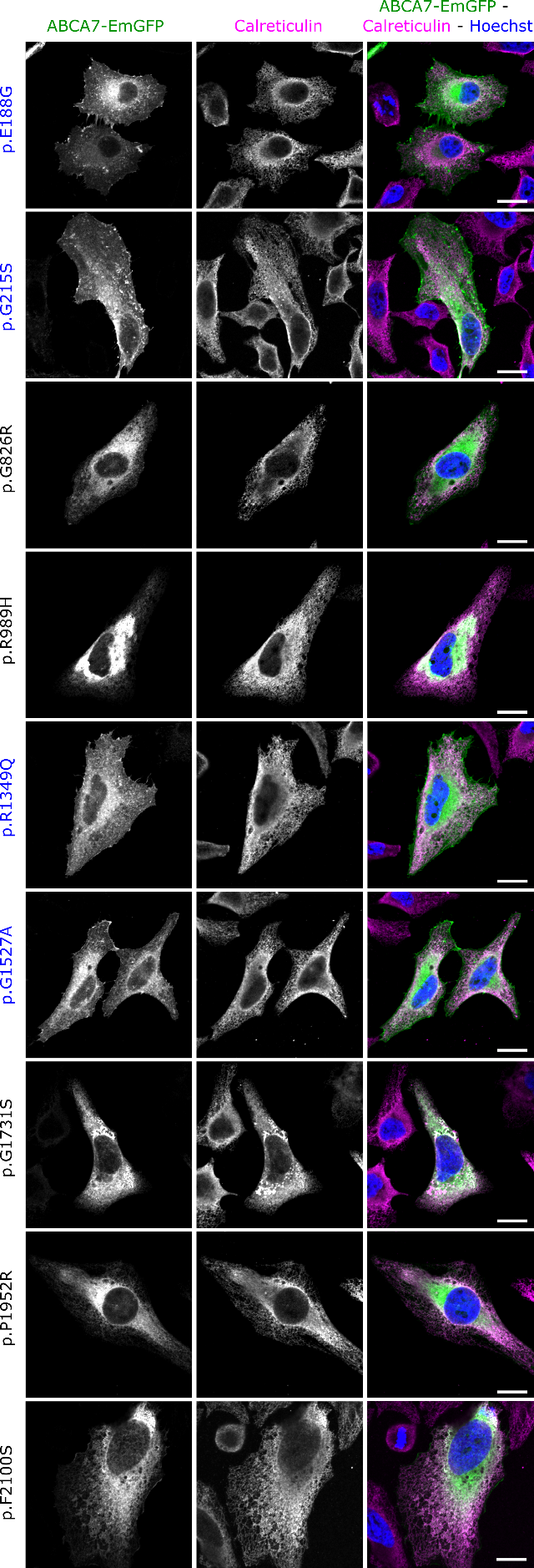
**

**Fig. S10** Subcellular localization of individual mutant and benign ABCA7-EmGFP constructs. Confocal microscopy images of HeLa cells transiently expressing ABCA7-EmGFP are shown. Predicted pathogenic mutants are indicated with black text color, predicted benign variants are indicated with blue text color. Calreticulin (magenta) was used as a marker for the ER to investigate colocalization between ABCA7 and the ER. Scale bars represent 20µm.

**REFERENCES**

1. Ye, J., et al., *Primer-BLAST: A tool to design target-specific primers for polymerase chain reaction.* BMC Bioinformatics, 2012. **13**(1): p. 134.

2. Weckx, S., et al., *novoSNP, a novel computational tool for sequence variation discovery.* Genome research, 2005. **15**(3): p. 436-442.

3. Li, H., *Minimap2: pairwise alignment for nucleotide sequences.* Bioinformatics, 2018. **34**(18): p. 3094-3100.

4. Edge, P. and V. Bansal, *Longshot enables accurate variant calling in diploid genomes from single-molecule long read sequencing.* Nature Communications, 2019. **10**(1): p. 4660.

5. Love, S., et al., *Development, appraisal, validation and implementation of a consensus protocol for the assessment of cerebral amyloid angiopathy in post-mortem brain tissue.* Am J Neurodegener Dis, 2014. **3**(1): p. 19-32.

6. Thal, D.R., et al., *Capillary cerebral amyloid angiopathy identifies a distinct APOE epsilon4-associated subtype of sporadic Alzheimer's disease.* Acta Neuropathol, 2010. **120**(2): p. 169-83.

7. Hyman, B.T., et al., *National Institute on Aging-Alzheimer's Association guidelines for the neuropathologic assessment of Alzheimer's disease.* Alzheimer's & dementia : the journal of the Alzheimer's Association, 2012. **8**(1): p. 1-13.

8. El Khoury, P., et al., *Identification of the first Tangier disease patient in Lebanon carrying a new pathogenic variant in ABCA1.* Journal of Clinical Lipidology, 2018. **12**(6): p. 1374-1382.

9. Landry, Y.D., et al., *ATP-binding Cassette Transporter A1 Expression Disrupts Raft Membrane Microdomains through Its ATPase-related Functions*.* Journal of Biological Chemistry, 2006. **281**(47): p. 36091-36101.

10. Vaughan, A.M., C. Tang, and J.F. Oram, *ABCA1 mutants reveal an interdependency between lipid export function, apoA-I binding activity, and Janus kinase 2 activation.* Journal of lipid research, 2009. **50**(2): p. 285-292.

11. Shroyer, N.F., et al., *Null Missense ABCR (ABCA4) Mutations in a Family with Stargardt Disease and Retinitis Pigmentosa.* Investigative Ophthalmology & Visual Science, 2001. **42**(12): p. 2757-2761.

12. Garces, F.A., J.F. Scortecci, and R.S. Molday, *Functional Characterization of ABCA4 Missense Variants Linked to Stargardt Macular Degeneration.* International journal of molecular sciences, 2020. **22**(1): p. 185.

13. Garces, F., et al., *Correlating the Expression and Functional Activity of ABCA4 Disease Variants With the Phenotype of Patients With Stargardt Disease.* Investigative ophthalmology & visual science, 2018. **59**(6): p. 2305-2315.

14. The UniProt, C., *UniProt: the universal protein knowledgebase in 2021.* Nucleic Acids Research, 2021. **49**(D1): p. D480-D489.

15. Sievers, F., et al., *Fast, scalable generation of high-quality protein multiple sequence alignments using Clustal Omega.* Molecular Systems Biology, 2011. **7**(1): p. 539.

16. Landrum, M.J., et al., *ClinVar: improving access to variant interpretations and supporting evidence.* Nucleic acids research, 2018. **46**(D1): p. D1062-D1067.

17. Kircher, M., et al., *A general framework for estimating the relative pathogenicity of human genetic variants.* Nat Genet, 2014. **46**(3): p. 310-5.
